# RIPK3–MLKL necroptotic signalling amplifies STING pathway and exacerbates lethal sepsis

**DOI:** 10.1002/ctm2.1334

**Published:** 2023-07-20

**Authors:** Xufei Zhang, Jie Wu, Qinjie Liu, Xuanheng Li, Yiyu Yang, Lei Wu, Xiuwen Wu, Yun Zhao, Jianan Ren

**Affiliations:** ^1^ Research Institute of General Surgery, Jinling Hospital, School of Medicine Southeast University Nanjing China; ^2^ Research Center of Surgery, BenQ Medical Center the Affiliated BenQ Hospital of Nanjing Medical University Nanjing China; ^3^ Research Institute of General Surgery, Jinling Hospital, Affiliated Hospital of Medical School Nanjing University Nanjing China

**Keywords:** inflammation, MLKL, RIPK3, sepsis, STING

## Abstract

**Backgrounds:**

The stimulator of interferon genes (STING) is an important driver in various inflammatory diseases.

**Methods and results:**

Here, we have demonstrated that inhibition of RIPK3 and MLKL dampens STING signaling, indicating that necroptosis may be involved in sustaining STING signaling. Furthermore, RIPK3 knockout in HT‐29 cells significantly suppressed STING signaling. Mechanistically, RIPK3 inhibits autophagic flux during STING activation. RIPK3 knockout inhibits STING signaling by intensifying STING autophagy. In contrast, MLKL regulates the STING pathway bidirectionally. MLKL deficiency enhances STING signaling, whereas suppression of MLKL‐mediated pore formation restricts STING signaling. Mechanistically, upon abrogating the pro‐necroptotic activity of MLKL, MLKL bound to activated STING is secreted to the extracellular space, where it restricts TBK1 and IRF3 recruitment. Targeting necroptotic signaling ameliorates STING activation during DMXAA‐induced intestinal injury and sepsis.

**Conclusions:**

These findings elucidate molecular mechanisms linking necroptosis to the STING pathway, and suggest a potential benefit of therapeutic targeting of necroptosis in STING‐driven inflammatory diseases.

## Graphical abstract

2. RIPK3 participates in inhibiting autophagy and sustains STING signalling.

3. Intrinsic MLKL bound to activated STING is secreted to the extracellular space, which is restrained by the pro‐necroptotic activity of MLKL.

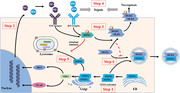

## 1 INTRODUCTION

Stimulator of interferon genes (STING) signalling mediates inflammation and cell death in recognition to cyclic dinucleotides or cytosolic DNA. Activated STING is trafficked from the endoplasmic reticulum to the Golgi, recruits TANK‐binding kinase 1 (TBK1) and interferon regulatory factor 3 (IRF3) and subsequently induces interferons (IFNs) and other cytokines.^1^, ^2^ Increasing evidence indicates that STING signalling is involved in cell death and autophagy.^3^ STING induction triggers autophagy, leading to STING degradation.^4^ Overactivation of STING signalling is associated with multiple inflammatory diseases, including inflammatory bowel disease, auto‐inflammatory diseases, sepsis and non‐alcoholic steatohepatitis.^5^, ^6^, ^7^, ^8^ It is thus important to understand targets that regulate the STING pathway.

Necroptosis is a form of programmed cell death triggered by multiple cytokines, death receptors and pattern‐recognition receptors.^9^ Receptor‐interacting protein kinase 3 (RIPK3) and mixed lineage kinase domain‐like protein (MLKL) are essential mediators of necroptosis. Upon activation, a necrosome complex is formed by interaction between RIPK3 and RIPK1. RIPK1 and RIPK3 are activated via autophosphorylation. Activated RIPK3 recruits and phosphorylates MLKL. Phosphorylated MLKL then executes necroptosis.^10^ Studies on RIPK3 and MLKL have largely been restricted to their roles in necroptosis. Nevertheless, increasing evidence indicates that RIPK3 and MLKL also exert non‐deadly functions. Necroptosis‐independent functions of RIPK3 have been revealed in autophagy, cell proliferation and mitochondrial metabolism.^11^ MLKL also executes necroptosis and regulates various biological processes (BPs), including autophagy, endosomal trafficking, extracellular vesicle generation, inflammasome activation and endoplasmic reticulum stress.^12^ Therefore, RIPK3 and MLKL may also regulate other signalling pathways during necroptosis.

Here, we found that different necroptotic signalling inhibitors remarkably restricted the STING pathway. Moreover, RIPK3 knockout in HT‐29 cells significantly suppressed STING signalling. MLKL deficiency enhanced STING signalling, whereas suppression of MLKL‐mediated pore formation restricted STING signalling. Both RIPK3 and MLKL play crucial roles in maintaining STING signalling activation. These findings suggest that RIPK3 and MLKL are novel therapeutic targets in STING‐mediated inflammatory diseases.

## 2 RESULTS

### 2.1 Necroptosis is not solely a downstream effect of STING signalling

Our previous studies have shown that STING activation can trigger necroptosis, thereby aggravating intestinal ischemia reperfusion injury.^13^ Moreover, STING induced necroptotic signalling in immune and nonimmune cells stimulated with various STING agonists (Figures S1A and B). However, blockade of RIPK1 or RIPK3 with necrostatin‐1 (Nec‐1) or GSK‐872 (GSK) significantly dampened STING and IRF3 phosphorylation levels in RAW264.7 cells under DMXAA stimuli (Figures S1C and D). We thus speculated that necroptosis may play a critical role in STING signalling. In addition, different necroptosis inhibitors remarkably suppressed a DMXAA‐activated IFN‐luciferase reporter in RAW264.7 cells with an IFN‐luciferase reporter construct (Figures S1E). Blocking necroptotic signalling also inhibited the induction of IFNβ and TNF‐α mRNA by DMXAA (Figures S1F).

MLKL is the terminal effector of RIPK1 and RIPK3. Currently, available MLKL inhibitors mainly disrupt MLKL‐mediated pore formation. We explored the effects of MLKL‐mediated membrane perturbation on STING signalling. Necrosulfonamide (NSA), a human MLKL inhibitor, was used in this study. Intriguingly, NSA significantly inhibited STING signalling and induction of IFNβ mRNA induced by ADU‐S100 (ADU), a human STING agonist, in THP‐1 cells (Figures S1H and I).

### 2.2 RIPK3 deficiency in HT‐29 cells ameliorates STING signalling

**FIGURE 1:**
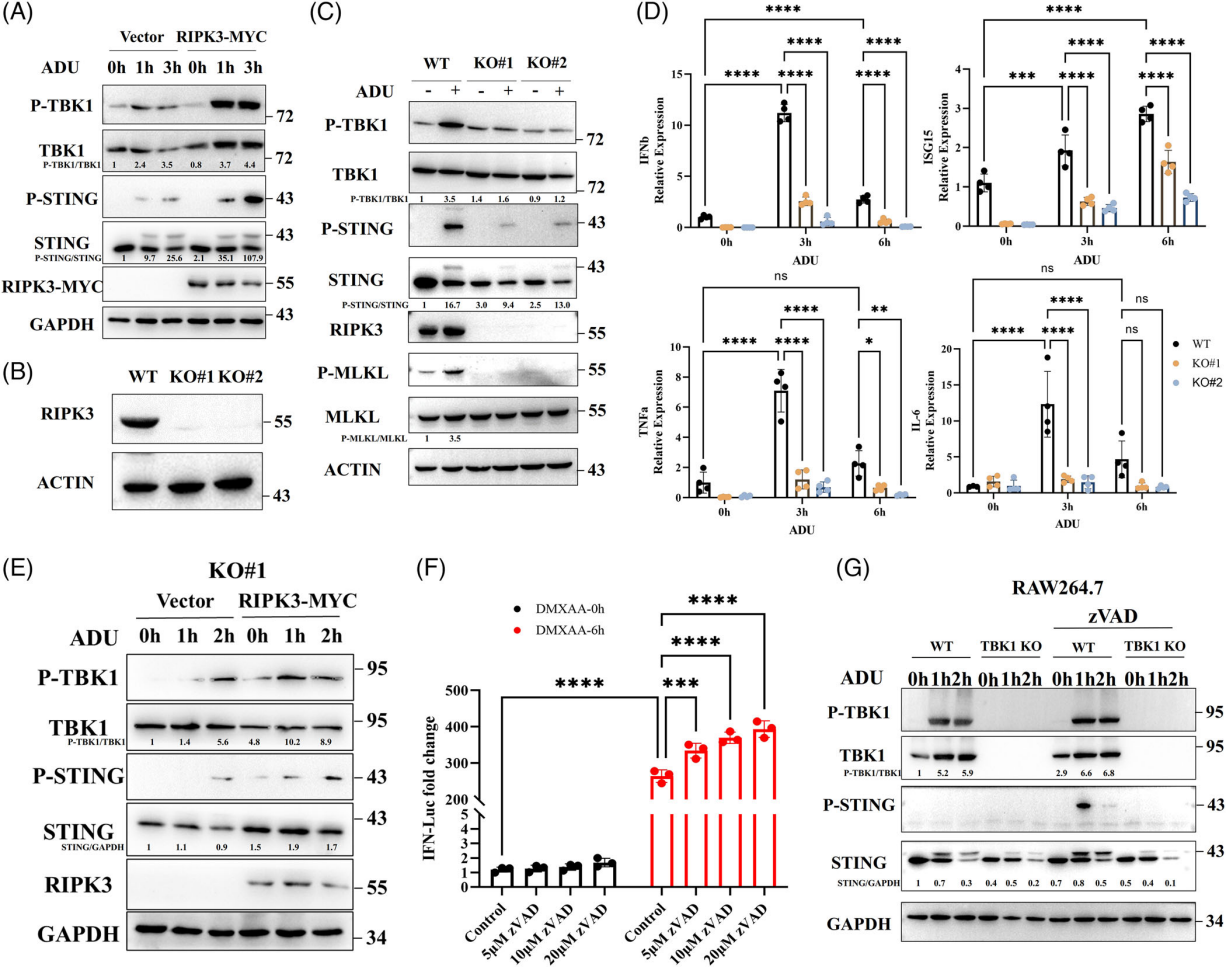
RIPK3 deficiency in HT‐29 cells restrains STING signalling. (A) Immunoblot analysis of ADU‐mediated STING signalling in HT‐29 cells transfected with RIPK3 encoding plasmids. The experiment was repeated at least three times. (B) Two RIPK3 knockout HT‐29 cell lines were selected by using the CRISPR/Cas9 system and immunoblot was conducted to verify RIPK3 knockout. (C) Western blot analysis of STING signalling in WT and RIPK3 knockout HT‐29 cells under ADU stimulation (27 µM). The experiment was repeated at least three times. (D) qPCR analysis of IFN‐β, ISG15, TNF‐α and IL‐6 in WT and RIPK3 knockout HT‐29 cells under ADU stimulation (27 µM). (E) Immunoblot analysis of ADU‐mediated STING signalling in RIPK3 knockout HT‐29 cells transfected with RIPK3 encoding plasmids. The experiment was repeated at least three times. (F) Assessment of DMXAA‐mediated IFN luciferase reporter activation in RAW264.3 cells stimulated with zVAD in a dose‐dependent manner. (G) Immunoblot analysis of STING signalling in WT and TBK1 knockout RAW264.7 cells treated with ADU (13.5 µM) and zVAD (30 µM). The experiment was repeated at least three times. Data were shown as the mean ± SD. *p < 0.05, **p < 0.01, ***p < 0.001, ****p < 0.0001.

RIPK3 is a central link in necroptotic signalling. To further determine the role of necroptosis in STING signalling, we transfected HT‐29 cells with a plasmid expressing RIPK3‐MYC. Upon stimulation with ADU, HT‐29 cells overexpressing RIPK3 showed enhanced activation of STING signalling (Figure 1A). To assess the role of RIPK3 in this process more precisely, we prepared two RIPK3 knockout HT‐29 cell lines (Figure 1B). As shown in Figure 1C, the RIPK3 knockout HT‐29 cell lines were not sensitised to ADU. Meanwhile, RIPK3 knockout significantly abrogated the expression of inflammatory cytokines (IFNβ, IL‐6 and TNF‐α) and IFN‐stimulated genes (ISG15) under ADU stimuli (Figure 1D). Expression of RIPK3 in RIPK3 knockout HT‐29 cell restored the phosphorylation of TBK1 and STING (Figure 1E).

RIPK3 activation is required for inhibition of caspase signalling. Z‐VAD‐FMK (zVAD), a pan‐caspase inhibitor, induces necrosome formation between RIPK1 and RIPK3, consequently activating RIPK3 and inducing necroptosis. We found that zVAD markedly stimulated a DMXAA‐activated IFN‐luciferase reporter in a dose‐dependent manner and enhanced STING phosphorylation (Figures 1F and G). Notably, zVAD significantly inhibited STING degradation in WT RAW264.7 cells (Figure 1G). However, zVAD did not affect STING degradation in RAW 264.7 cells with TBK1 knockout, suggesting that RIPK3 is involved in STING signalling after STING recruits TBK1. In addition, inhibition of RIPK1 with nec‐1 or MLKL depletion did not reverse the inhibitory effect of RIPK3 knockout on ADU‐induced STING signalling (Figures S2A and B). Collectively, these results indicated that RIPK3 plays a crucial role in maintaining STING activation.

### 2.3 RIPK3 sustains activation of STING signalling by restraining STING autophagy

**FIGURE 2:**
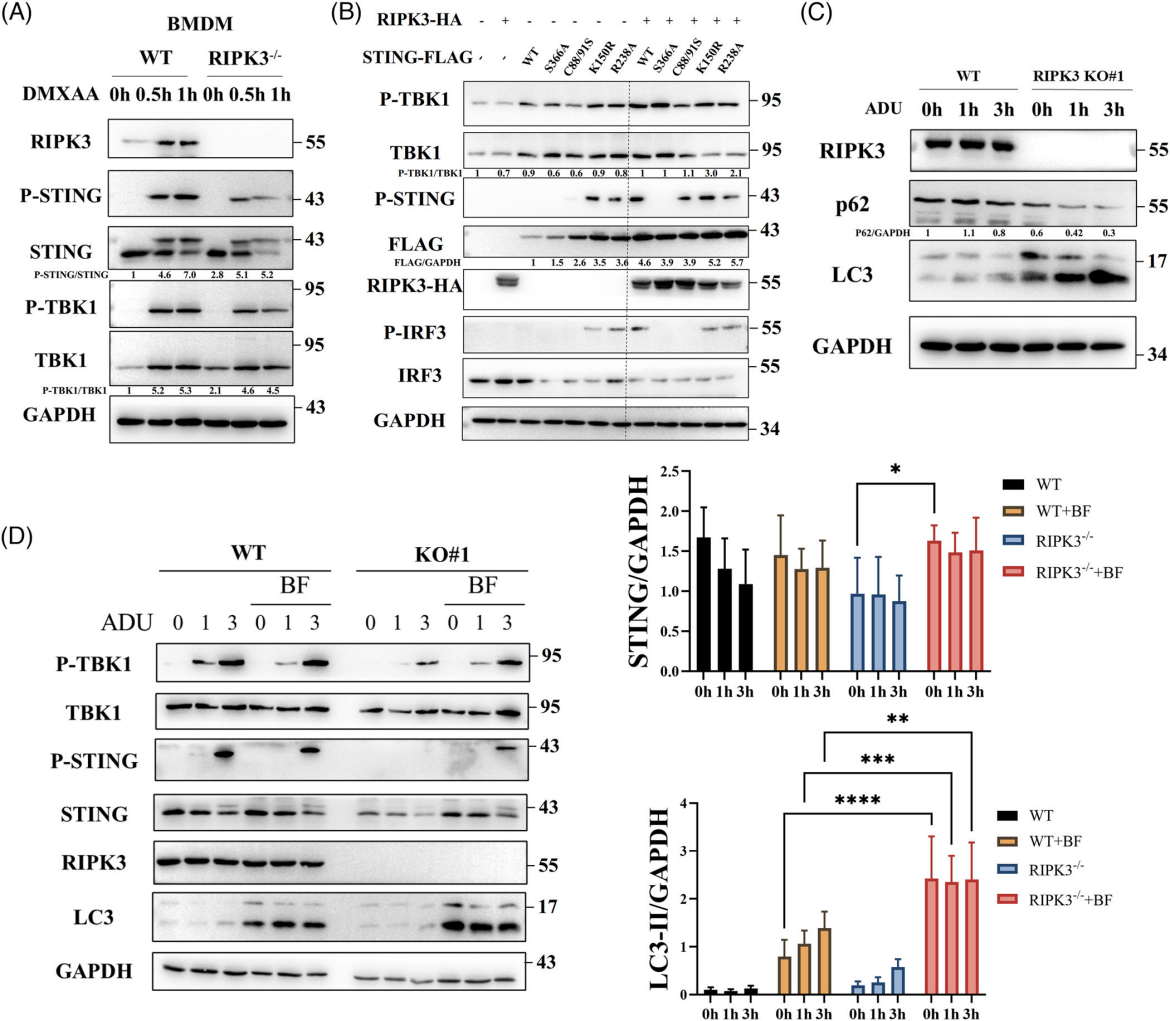
RIPK3 sustains activation of STING signalling by restraining the autophagy of STING. (A) Western blot analysis of STING signalling in BMDM from WT and RIPK3^−/−^ mice after stimulation with DMXAA (50 µg/mL). The experiment was repeated at least three times. (B) HEK293T cells were transfected with various STING mutant plasmid and RIPK3 plasmid. Western blot was performed to analyse STING signalling. The experiment was repeated at least three times. (C) Immunoblot analysis of autophagy‐related protein (p62 and LC3) in WT and RIPK3 knockout HT‐29 cells stimulated with ADU (27 µM). The experiment was repeated at least three times. (D) Immunoblot analysis of STING signalling in WT and RIPK3 knockout HT‐29 cells stimulated with ADU (27 µM) and bafilomycin A1 (100 nM). Quantitative analysis of STING and LC3‐II was performed. The experiment was repeated at least three times. Data were shown as the mean ± SD. *p < 0.05, **p < 0.01, ***p < 0.001, ****p < 0.0001.

To further validate the role of RIPK3 in STING signalling, bone‐marrow‐derived macrophages (BMDMs) were obtained from WT and RIPK3^−/−^ mice. RIPK3 deficiency in BMDMs significantly restricted STING signalling activation under DMXAA stimulation (Figure 2A). To explore internal regulatory mechanisms, we constructed a series of functional STING mutant plasmid, including S366A, C88/91S, K150R and R238A. STING–S366A mutants possess restricted ability to activate IRF3, STING–C88/91S mutants eliminate STING palmitoylation, STING–K150R mutants abolish STING ubiquitination and STING–R238A mutants abolish its ability to induce autophagy.^4^, ^14^, ^15^, ^16^ Interestingly, STING expression was similar to that of STING–R238A mutants upon transfection with RIPK3, while expression of WT STING transfected alone was much lower than that of STING–R238A mutants due to autophagic STING degradation (Figure 2B), indicating that RIPK3 probably regulates STING autophagy. We found significantly decreased levels of P62 and increasing levels of LC3‐II in RIPK3 knockout HT‐29 cells during STING activation (Figure 2C). Simultaneously, bafilomycin A1 (BF), an inhibitor of autophagy, significantly enhanced STING phosphorylation in RIPK3 knockout HT‐29 cells (Figure 2D). Moreover, quantitative analysis showed that BF significantly increased STING in RIPK3 knockout HT‐29 cells. After treatment with BF, increased levels of LC3‐II were observed in RIPK3 knockout HT‐29 cells compared with those in WT HT‐29 cells, indicating that RIPK3 knockout enhances STING autophagy (Figure 2D). We also found that activated STING interacted with LC3 in RIPK3 KO HT‐29 cells, whereas activated STING clustered around the nucleus in WT HT‐29 cells (Figure S3A).

### 2.4 The kinase domain of RIPK3 regulates STING autophagy

**FIGURE 3:**
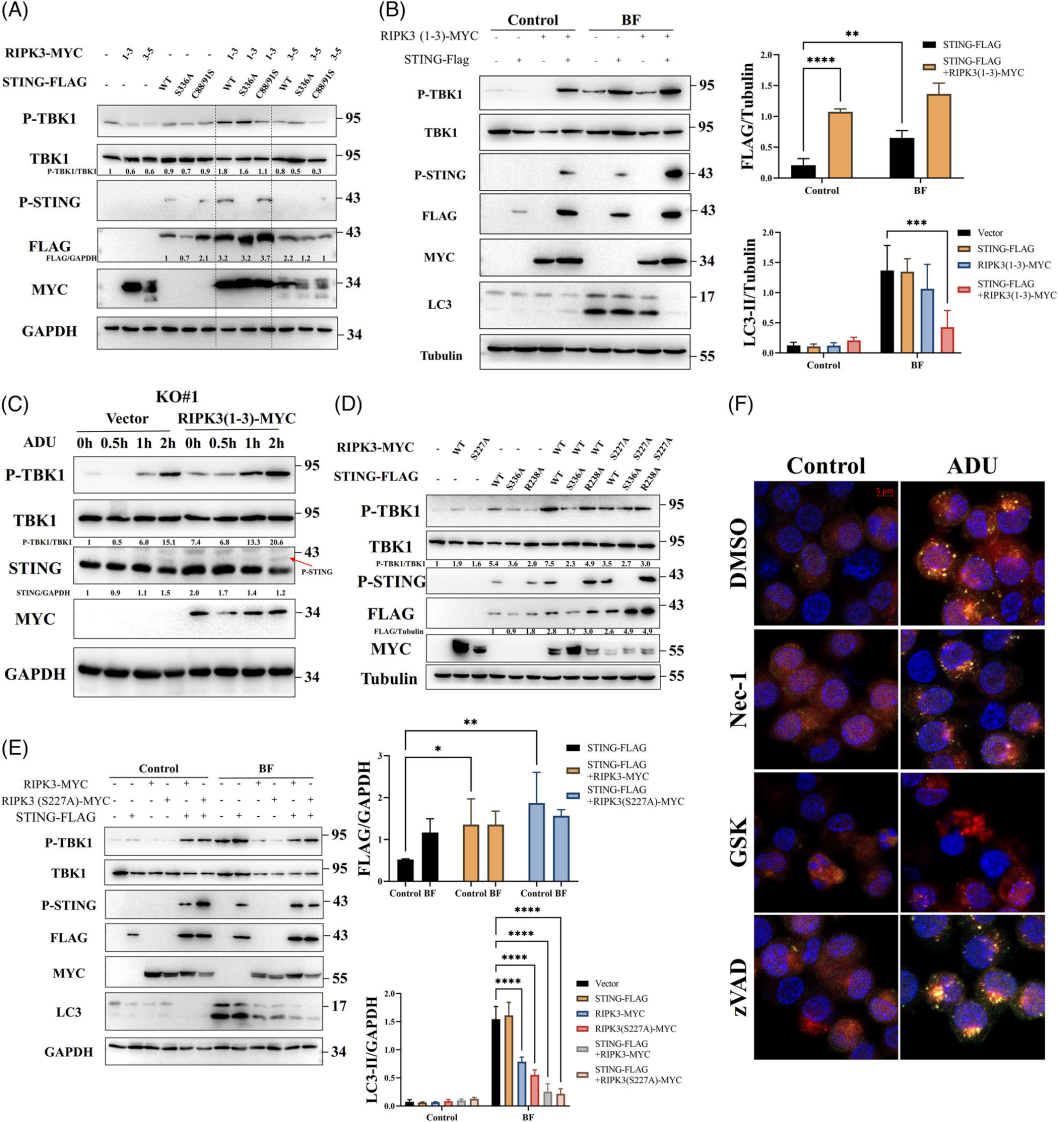
The kinase domain of RIPK3 regulates the autophagy of STING. (A and B) HEK293T cells were transfected with several RIPK3 mutant plasmid and STING plasmid. Western blot was conducted to analyse STING signalling. Bafilomycin A1 (100 nM) was used for analysis of autophagy flux. Quantitative analysis of STING and LC3‐II was performed. The experiment was repeated at least three times. (C) Immunoblot analysis of STING signalling in RIPK3 knockout HT‐29 cells transfected with the kinase domain of RIPK3 under ADU stimulation (27 µM). The experiment was repeated at least three times. (D and E) HEK293T cells were transfected with several RIPK3 mutant plasmid and STING mutant plasmid. Western blot was conducted to analyse STING signalling. Bafilomycin A1 (100 nM) was used for analysis of autophagy flux. Quantitative analysis of STING and LC3‐II was performed. The experiment was repeated at least three times. (F) Confocal microscopy analysis of autophagic flux in RAW 264.7 cells with GFP–RFP‐tagged LC3 treated with ADU (13.5 µM), Nec‐1 (45 µM), GSK (5 µM) and zVAD (30 µM). Nuclear DNA was labelled using DAPI. Data were shown as the mean ± SD. *p < 0.05, **p < 0.01, ***p < 0.001, ****p < 0.0001.

Next, we wondered which functional domain of RIPK3 is implicated in STING autophagy. RIPK3 contains a kinase domain and a RIP homotypic interaction motif (RHIM) domain.^10^ We constructed two truncated mutant RIPK3 plasmids containing amino acid residues 1−310 (1–3) and amino acid residues 311−518 (3–5). RIPK3 (1–3) contains a kinase domain, whereas RIPK3 (3–5) contains the RHIM domain. After transfecting STING plasmid and the truncated mutant plasmid of RIPK3 into HEK293T cells, we found that the kinase domain of RIPK3 significantly reinforced STING signalling, accompanied by increased STING protein levels (Figure 3A), indicating that kinase activity of RIPK3 regulates STING autophagy. We also observed that co‐expression of STING and RIPK3 (1–3) resulted in lower LC3‐II levels than expression of STING or RIPK3 (1–3) alone after BF treatment (Figure 3B), suggesting that the interaction between STING and RIPK3 play a critical role in decreasing autophagic flux. Moreover, re‐expression of the kinase domain of RIPK3 restored STING signalling in RIPK3 knockout HT‐29 cells (Figure 3C).

RIPK3 pro‐necroptotic activity depends on its kinase domain. To characterise RIPK3 pro‐necroptotic activity, we constructed RIPK3–S227A mutant plasmids, which abolished its ability to mediate necroptosis. We observed that both WT RIPK3 and the RIPK3–S227A mutant enhanced STING signalling (Figure 3D). We also analysed autophagic flux. After BF treatment, co‐expression of STING with WT RIPK3 or RIPK3–S227A decreased LC3‐II levels compared with the vector group or single expression of STING (Figure 3E).

To identify the role of RIPK3 kinase activity of in autophagic flux, we used RAW 264.7 cells with GFP–RFP‐tagged LC3. As shown in Figure 3F, inhibition of the kinase activity of RIPK3 with Nec‐1 and GSK reduced the accumulation of yellow dots under ADU stimulation, whereas inducing the kinase activity of RIPK3 with zVAD enhanced the accumulation of yellow dots. These data cumulatively suggest that the RIPK3 kinase domain plays an essential role in STING signalling by restraining STING autophagy.

### 2.5 Inhibition of MLKL‐mediated membrane perturbation restrains STING signalling

**FIGURE 4:**
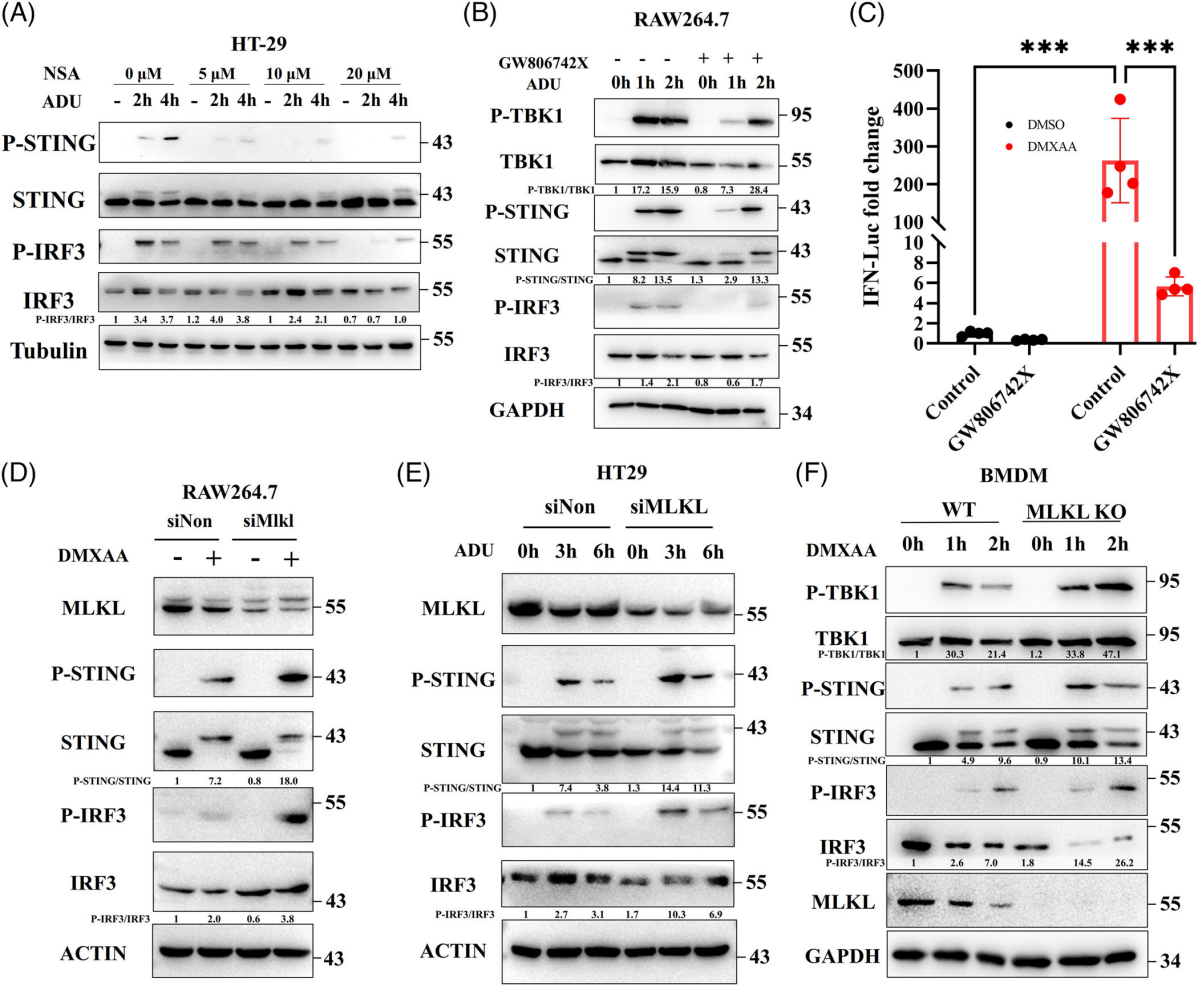
Inhibition of MLKL‐mediated membrane perturbation restrains STING signalling. (A) Western blot analysis of STING signalling in HT‐29 cells after stimulation with ADU (27 µM) and NSA in a dose‐dependent manner. The experiment was repeated at least three times. (B) Immunoblot analysis of STING signalling in RAW264.7 cells after stimulation with ADU (13.5 µM) and GW806742X (10 µM). The experiment was repeated at least three times. (C) Assessment of DMXAA‐mediated IFN luciferase reporter activation in RAW264.3 cells stimulated with GW806742X (10 µM). (D and E) Immunoblot analysis of STING signalling in MLKL knockdown RAW264.7 cells or HT‐29 cells treated with DMXAA (50 µg/mL) or ADU (27 µM). The experiment was repeated at least three times. (F) BMDMs were extracted from WT and MLKL^−/−^ mice and were stimulated with DMXAA (50 µg/mL). Western blot was conducted to detect STING signalling. The experiment was repeated at least three times. Data were shown as the mean ± SD. *p < 0.05, **p < 0.01, ***p < 0.001, ****p < 0.0001.

The pro‐necroptotic activity of RIPK3 is dispensable for STING autophagy (Figure 3E). However, NSA weakens STING signalling in immune and nonimmune cells (Figures S1H–I and 4A), indicating a potential role of MLKL in STING signalling. NSA also decreased the expression of inflammatory cytokines and IFN‐stimulated genes (Figure S4A). To exclude the effects of NSA on STING, GW806742X, a murine MLKL inhibitor, was employed. Consistent with the effects of NSA, GW806742X suppressed STING, TBK1 and IRF3 phosphorylation (Figure 4B). Moreover, GW806742X significantly attenuated DMXAA‐activated IFN‐luciferase reporter expression (Figure 4C). Mechanistically, both NSA and GW806742X bind to MLKL, block MLKL oligomerisation and retard its translocation to the membrane. Therefore, we speculated that the oligomerisation or translocation of MLKL to membranes is necessary for STING activation. Interestingly, silencing or knockout of MLKL facilitated STING signalling (Figures 4D–F). Collectively, our data indicate that MLKL regulates STING signalling, in contrast to RIPK3.

### 2.6 MLKL overexpression prominently inhibits STING signalling

**FIGURE 5:**
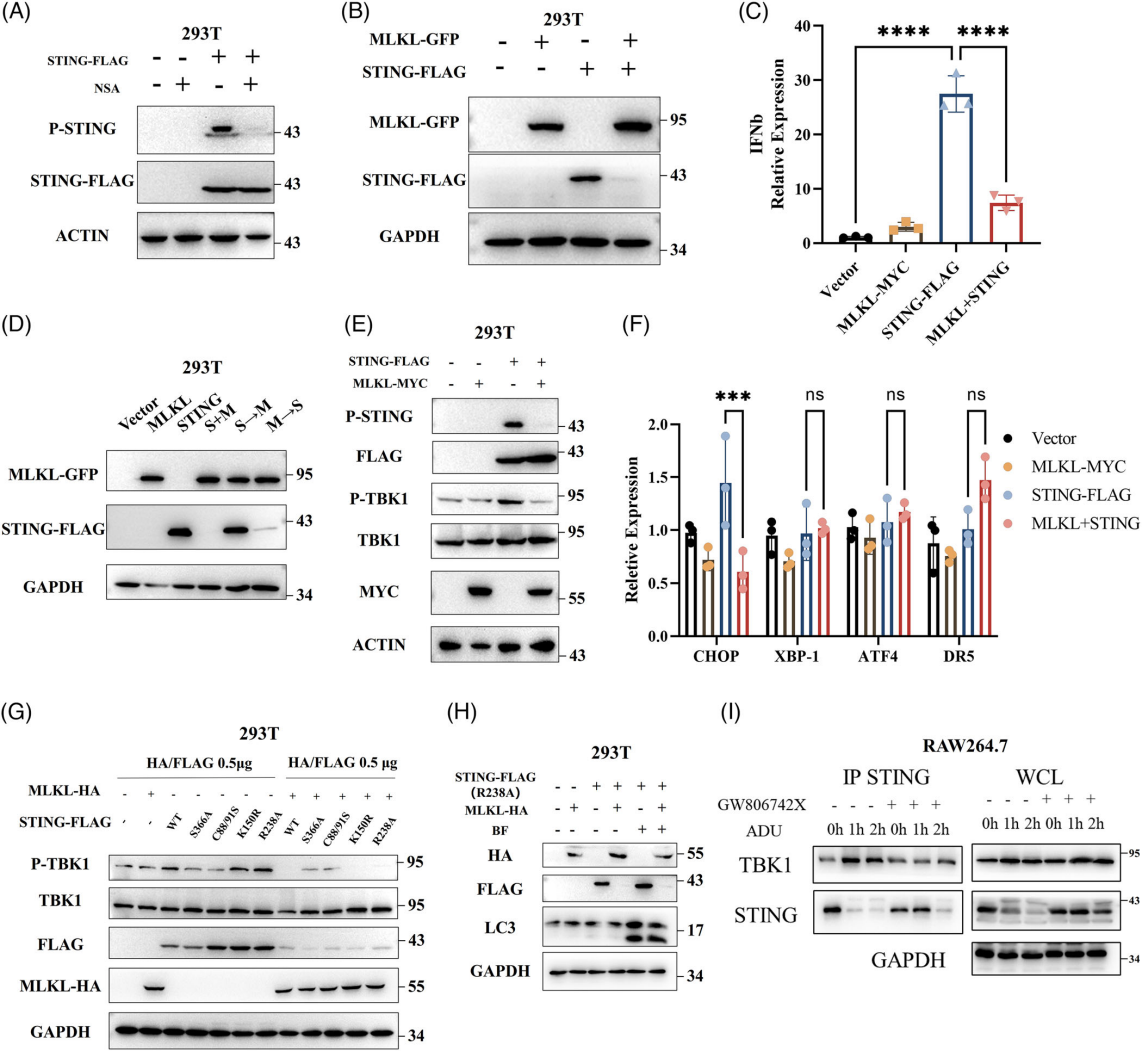
Overexpression of MLKL prominently inhibits STING signalling. (A) HEK293T cells were transfected with STING plasmid and treated with NSA (10 µM). Western blot was performed to detect STING signalling. The experiment was repeated at least three times. (B) Lysates from HEK293T cells transfected with STING plasmid and MLKL plasmid were probed for the indicated protein. The experiment was repeated at least three times. (C) qPCR analysis of IFNβ in HEK293T cells transfected with STING plasmid and MLKL plasmid. (D) STING plasmid and MLKL plasmid were co‐expressed in HEK293T cells by simultaneous transfection or separate transfection. Then, lysates were probed for the indicated protein. The experiment was repeated at least three times. (E) MLKL plasmid was transfected after STING plasmid in HEK293T cells. Then, lysates were probed for the indicated protein. The experiment was repeated at least three times. (F) qPCR analysis of ER stress markers (CHOP, XBP1, ATF4 and DR5) in HEK293T cells co‐expressed with STING plasmid and MLKL plasmid. (G) HEK293T cells were simultaneously transfected with various STING mutant plasmid and MLKL plasmid. Western blot was performed to analyse STING signalling. The experiment was repeated at least three times. (H) HEK293T cells were simultaneously transfected with STING–R238A mutants and MLKL plasmid and then stimulated with bafilomycin A1 (100 nM). The experiment was repeated at least three times. (I) Co‐immunoprecipitation of TBK1 with anti‐STING antibody and immunoblot analysis in RAW264.7 cells stimulated with ADU (13.5 µM) and GW806742X (10 µM). The experiment was repeated at least three times. Data were shown as the mean ± SD. *p < 0.05, **p < 0.01, ***p < 0.001, ****p < 0.0001.

Further experiments suggested NSA reduced STING phosphorylation in HEK293T cells transfected with STING plasmid (Figure 5A). To define the role of MLKL in STING signalling, we co‐expressed FLAG tagged STING and GFP tagged MLKL. Remarkably, STING was absent and the expression of IFNβ mRNA also was attenuated under MLKL transfection (Figures 5B and C). Next, we changed transfection strategies, including simultaneous transfection and separate transfection (4 h interval). Simultaneous transfection of STING and MLKL (S + M) was similar to STING transfection after MLKL transfection (M→S), whereas MLKL transfection after STING transfection (S→M) did not affect STING expression (Figure 5D). MLKL inhibited STING expression in a concentration‐dependent manner (Figure S5A). When the MLKL plasmid was transfected after the STING plasmid, MLKL still inhibited phosphorylation of STING signalling (Figure 5E), indicating that MLKL mainly restricts the presence of activated STING in cells.

As previously reported, necroptosis is associated with ER stress.^17^ To determine whether MLKL overexpression affects the synthesis of STING protein by inducing ER stress, we detected ER stress markers, including CHOP, XBP1, ATF4 and DR5. ER stress did not significantly increase after simultaneous transfection with STING or MLKL (Figure 5F). Next, we co‐expressed MLKL with several STING mutants in HEK293T cells. None of the STING mutants were successfully expressed in conjunction with MLKL transfection, including the STING–R238A mutants (Figure 5G). Inhibition of autophagy by BF did not alter the inhibitory effect of MLKL on STING (Figure 5H), indicating that autophagy is dispensable for STING signalling in such a condition. Intriguingly, the MLKL deletion mutant plasmid did not affect STING expression (Figure S5B), suggesting that the effect of MLKL on STING depends on the overall function of MLKL. To determine where MLKL affects STING during activated STING trafficking, STING immunoprecipitation was performed. Immunoprecipitation results showed that TBK‐1 recruitment to STING was remarkably decreased upon inhibition of MLKL‐mediated membrane perturbation (Figure 5I), indicating that MLKL regulates STING before it recruits TBK1.

### 2.7 MLKL binding to activated STING promotes extracellular secretion of the complex upon abrogating the pro‐necroptotic activity of MLKL

**FIGURE 6:**
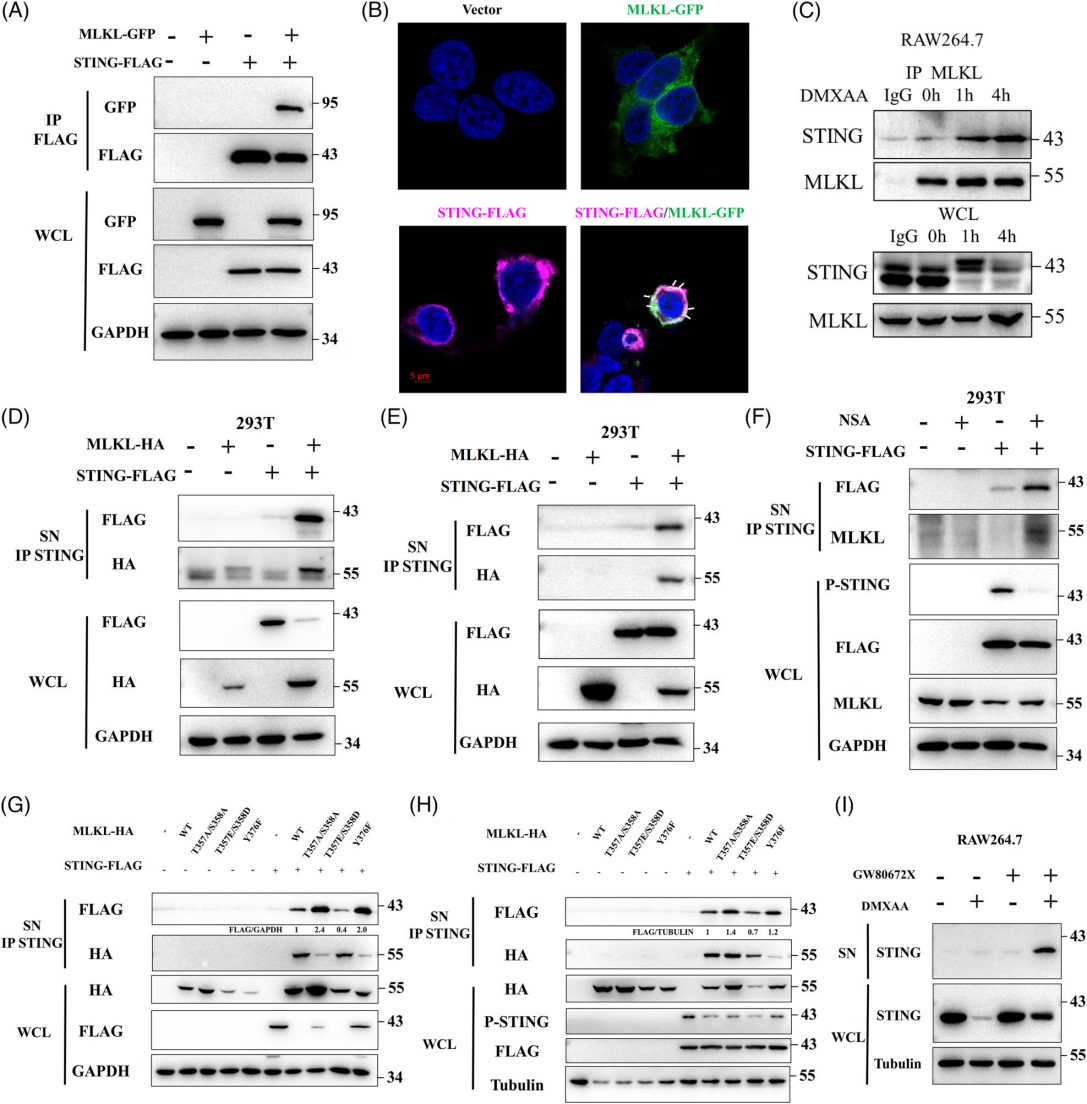
MLKL binding to activated STING is secreted outside cells upon abrogating the pro‐necroptotic activity of MLKL. (A) MLKL plasmid was expressed in HEK293T cells after STING plasmid at 4 h interval. The cell lysates were immunoprecipitated using anti‐FLAG antibody and then analysed by immunoblotting. The experiment was repeated at least three times. (B) Confocal microscopy analysis of STING and MLKL in HEK293T cells. Nuclear DNA was labelled using DAPI. White arrowheads point to colocalisation of STING and MLKL. (C) RAW264.7 cells were DMXAA (50 µg/mL). The cell lysates were immunoprecipitated using anti‐MLKL antibody and then analysed by immunoblotting. The experiment was repeated at least three times. (D) STING plasmid and MLKL plasmid were simultaneously expressed in HEK293T cells. The supernatant was immunoprecipitated using anti‐STING antibody and then analysed by immunoblotting. The experiment was repeated at least three times. (E) MLKL plasmid was expressed in HEK293T cells after transfection of STING plasmid at 4 h interval. The supernatant was immunoprecipitated using anti‐STING antibody and then analysed by immunoblotting. The experiment was repeated at least three times. (F) HEK293T cells were transfected with STING plasmid and then stimulated with NSA (10 µM). The supernatant was immunoprecipitated using anti‐STING antibody and then analysed by immunoblotting. The experiment was repeated at least three times. (G) STING plasmid and several MLKL mutant plasmid were simultaneously expressed in HEK293T cells. The supernatant was immunoprecipitated using anti‐STING antibody and then analysed by immunoblotting. (H) Several MLKL mutant plasmid was expressed in HEK293T cells after transfection of STING plasmid at 4 h interval. The supernatant was immunoprecipitated using anti‐STING antibody and then analysed by immunoblotting. (I) RAW 264.7 cells were treated with DMXAA (50 µg/mL) and GW806742X (10 µM). Protein in the supernatant was purified by using centrifugal filter and then analysed by immunoblotting. The experiment was repeated at least three times.

Further experiments demonstrated that STING transfected into HEK293T cells bound endogenous and exogenous MLKL (Figures 6A and S6A) and that MLKL associated with the CBD domain of STING (Figure S6B). Confocal microscopy revealed subcellular co‐localisation of MLKL and STING (Figure 6B). Moreover, nature immunoprecipitation revealed that STING interacts with MLKL under DMXAA stimulation in RAW264.7 cells (Figure 6C).

Previous studies verify MLKL facilitates formation of extracellular vesicles and enhances the release of related proteins.^18^ We thus speculated that the binding of MLKL to STING induces its release. To test this hypothesis, we used an antibody of STING and purified the protein from the supernatant using immunoprecipitation. Surprisingly, MLKL transfected into HEK293T cells bound STING and induced the release of STING into the supernatant regardless of simultaneous or separate transfection (Figures 6D and E). Moreover, inhibition of the pro‐necroptotic activity of MLKL resulted in the release of STING from the cells (Figure 6F). To investigate the internal regulatory mechanisms, we constructed a series of functional MLKL mutant plasmids, including T357A/S358A, T357E/S358D and Y376F. The MLKL–T357A/S358A and MLKL–Y376F mutants suppress the pro‐necroptotic activity of MLKL, whereas the MLKL–T357E/S358D mutant promotes its pro‐necroptotic activity. We demonstrated that all MLKL mutants induced STING secretion, but the MLKL–T357A/S358A and MLKL–Y376F mutants more strongly induced STING secretion (Figures 6G and H). Centrifugal filter was used to concentrate the supernatant. It was found that GW806742X promoted STING release in RAW264.7 cells under DMXAA stimulation (Figure 6I). MLKL appeared to be more sensitive to activated STING because administration of GW806742X alone did not affect STING. Collectively, these findings confirm that abrogating the pro‐necroptotic activity of MLKL is a target for restricting STING signalling.

### 2.8 Mice deficient in RIPK3 abrogate DMXAA‐induced destruction of the intestinal mucosa

**FIGURE 7:**
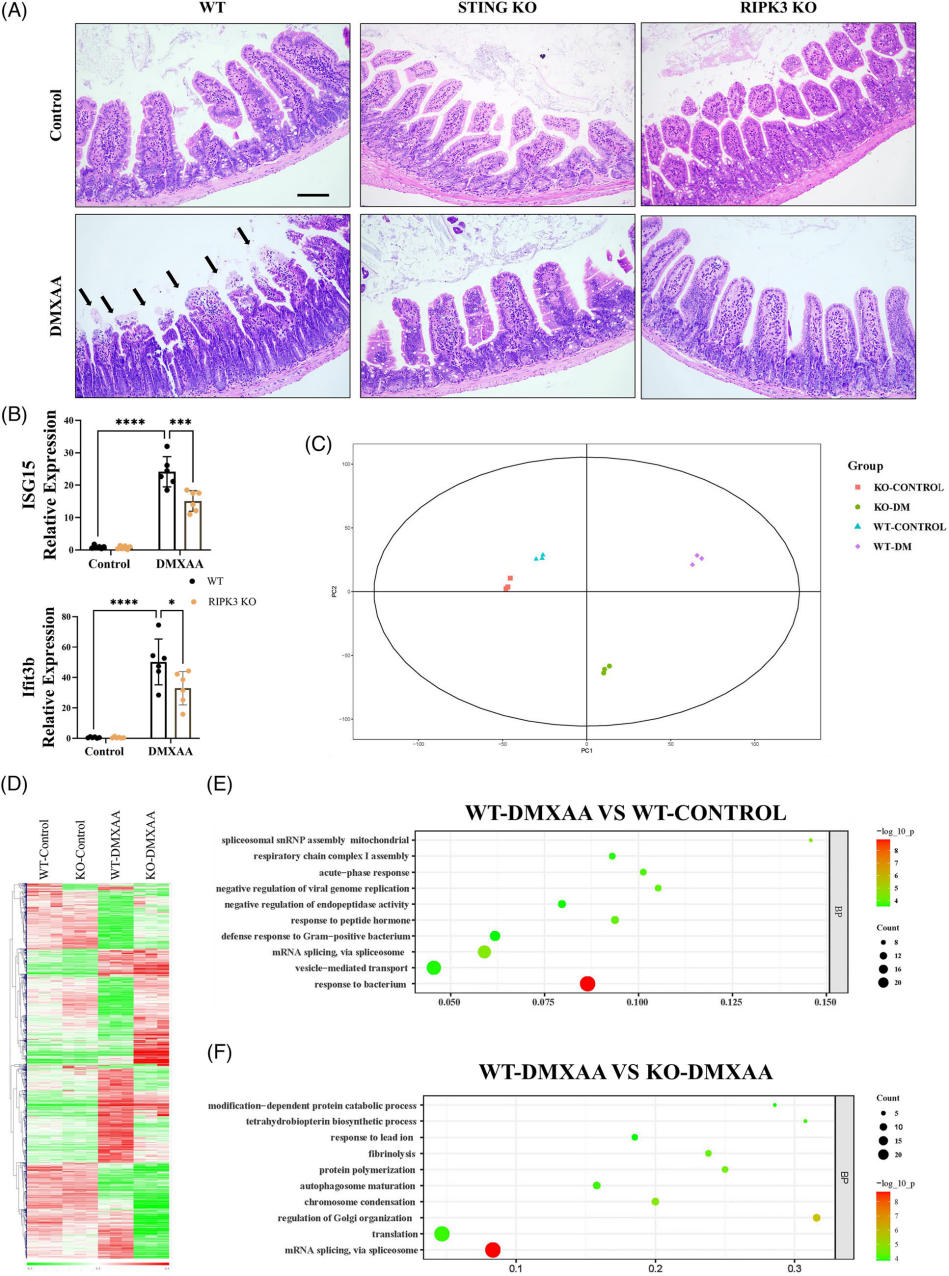
RIPK3 knockout abrogates DMXAA‐induced intestinal injury. (A) Representative images of intestinal H&E staining in WT, STING^−/−^ and RIPK3^−/−^ mice after DMXAA (25 mg/kg) injection for 6 h. Black arrowheads point to destruction of the intestinal mucosa. (B) qPCR analysis of interferon‐stimulated genes (ISG15 and Ifit3b) in small intestines of mice. (C) Principal component analysis (PCA) of protein in which input samples are clustered in the small intestines from WT and RIPK3^−/−^ mice. (D) Heatmap showing the differential expressed protein upon DMXAA stimulation from WT and RIPK3^−/−^ mice. (E) Gene Ontology (GO) showing significant pathways between WT‐Control and WT‐DMXAA. (F) GO showing significant pathways between WT‐DMXAA and RIPK3^−/−^‐DMXAA. The abscissa represents the enrichment factor of each GO item (richFator ≤ 1). The colour of bubbles represents the significance of enriched GO items based on Fisher's Exact Test. The bubble size indicates the number of differentially expressed proteins involved in the GO entry. Scale bars = 0.5 µm. Data were shown as the mean ± SD. *p < 0.05, **p < 0.01, ***p < 0.001, ****p < 0.0001.

To determine the role of necroptotic signalling in STING‐mediated tissue damage, a murine STING agonist (DMXAA) was injected intraperitoneally. Notably, treatment with DMXAA caused the destruction of the intestinal mucosa (Figure 7A). Deletion of STING in mice completely alleviated DMXAA‐induced intestinal injury, indicating that STING is necessary for DMXAA‐induced intestinal injury (Figure 7A). Moreover, RIPK3 knockout also abrogated DMXAA‐induced intestinal injury (Figure 7A). After DMXAA treatment, the mRNA levels of IFN‐stimulated genes (ISG15 and Ifit3b) in the small intestine of WT mice were elevated, whereas those in RIPK3^−/−^ mice decreased (Figure 7B). To distinguish the intrinsic changes in DMXAA‐treated intestines in WT and RIPK3^−/−^ mice, proteome profiling of intestines was performed. Principal component analysis (PCA) demonstrated that the proteome of the replicates in each group clustered tightly, indicating good reproducibility of measurements (Figure 7C and Table S1). A heatmap of DMXAA‐induced proteins sorted by differences between WT and RIPK3^−/−^ intestines confirmed that RIPK3 was required for a significant proportion of the post‐transcriptional responses induced by DMXAA (Figure 7D). Gene Ontology (GO) analysis revealed significant pathways for BP associated DMXAA‐dependent protein regulation with respiratory chain complex I assembly, acute‐phase response and vesicle‐mediated transport (Figure 7E). Furthermore, significant pathways for BP between WT and RIPK3^−/−^ intestines treated with DMXAA included modification‐dependent protein catabolic process, fibrinolysis, protein polymerisation, autophagosome maturation and regulation of Golgi organisation (Figure 7F). Together, proteome profiling indicated that STING activation is associated with vesicle‐mediated transport and that RIPK3 plays a pivotal role in the regulation of autophagosome maturation during STING activation.

### 2.9 Inhibition of necroptotic signalling protects against sepsis‐induced multiple organ dysfunction and systemic inflammation

**FIGURE 8:**
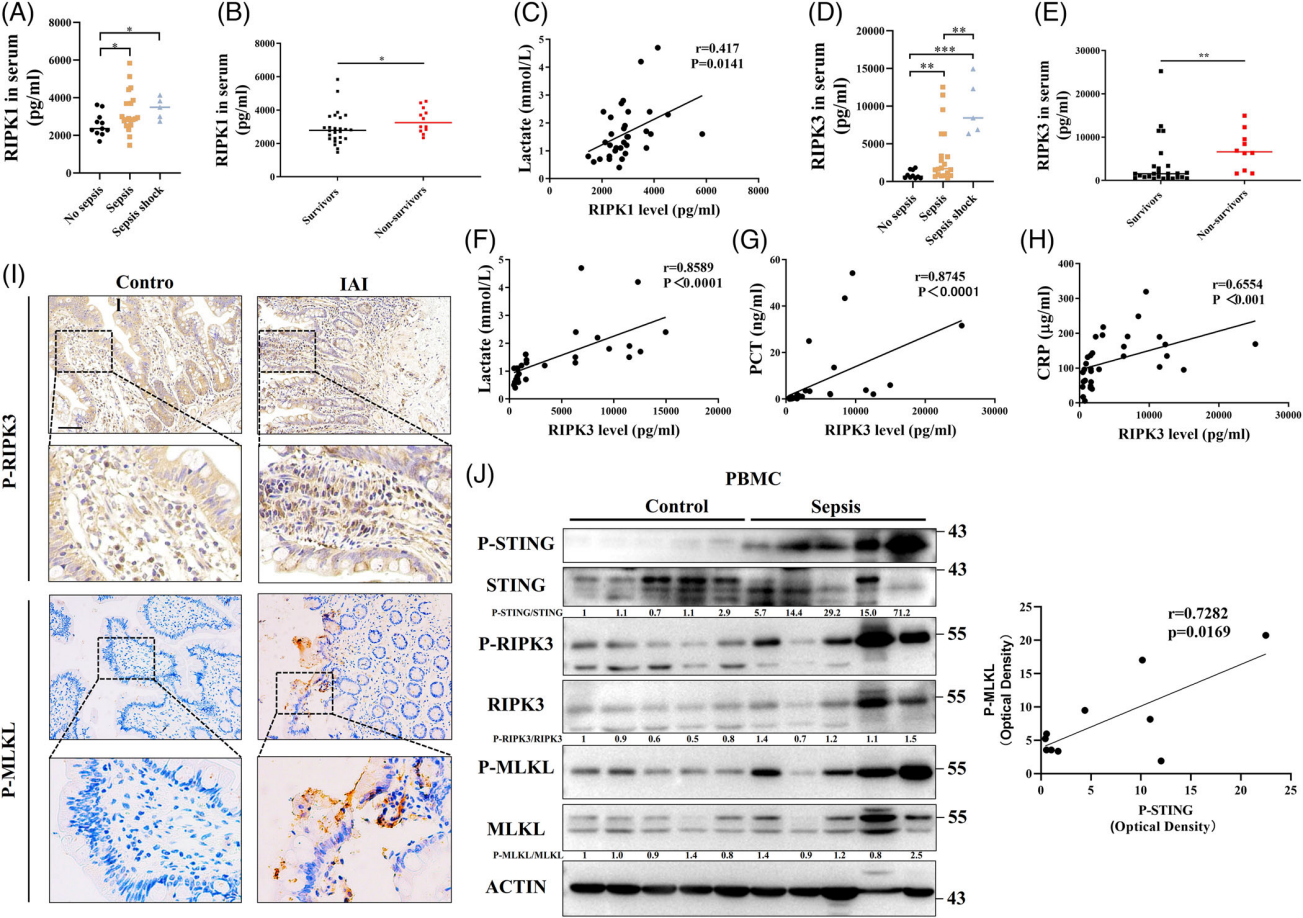
Enhanced necroptosis is associated with tissue damage, inflammation and STING signalling in sepsis. (A) Circulating RIPK1 in patients with no sepsis, sepsis and septic shock was analysed through enzyme‐linked immunosorbent assay (ELISA). (B) Circulating RIPK1 was compared between survivors and non‐survivors. (C) Correlation analysis between lactate and circulating RIPK1 was performed. (D) Circulating RIPK3 in patients with no sepsis, sepsis and septic shock was analysed through ELISA. (E) Circulating RIPK3 was compared between survivors and non‐survivors. (F–H) circulating lactate, PCT and CRP were recorded. Correlation analysis was performed. (I) Intestinal mucosae were collected from healthy control and intra‐abdominal infection (IAI) patients. Intestinal IHC staining for p‐RIPK3 and p‐MLKL was conducted. (J) Peripheral blood mononuclear cells (PBMC) were collected from healthy volunteers and patients with IAI. Western blot was performed to detect STING signalling and necroptotic signalling. Correlation analysis between p‐MLKL and p‐STING was conducted.

Sepsis is identified as life‐threatening organ dysfunction and leads to high mortality.^19^, ^20^ Our previous study found that STING is involved in sepsis‐related organ dysfunction and systemic inflammation.^8^ In this study, we also found that RIPK1 and RIPK3, which are key proteins involved in necroptosis, were significantly upregulated in patients with sepsis and septic shock (Figures 8A and D). To some extent, higher RIPK1 and RIPK3 levels in the circulation predicted in‐hospital death (Figures 8B and E). Lactate is a biomarker of cell and tissue damage, and a serum lactate level greater than 2 mmol/L is one clinical criterion for identifying septic shock.^19^ Circulating RIPK1 and RIPK3 levels correlated significantly with circulating lactate levels (Figure 8C and F). Moreover, RIPK3 in the serum positively correlated with circulating infection and inflammatory indicators (PCT and CRP) (Figures 8G and H). Compared with control patients, there was significantly increased phosphorylation of RIPK3 and MLKL in the intestines with intra‐abdominal infection (IAI) (Figure 8I). Peripheral blood mononuclear cells (PBMCs) were also isolated from healthy controls and patients with sepsis. Western blotting demonstrated that STING and necroptotic signalling were remarkably increased in sepsis and there was a positive correlation between p‐MLKL and p‐STING (Figure 8J). Thus, synergistic STING and necroptotic signalling contribute to sepsis.

**FIGURE 9:**
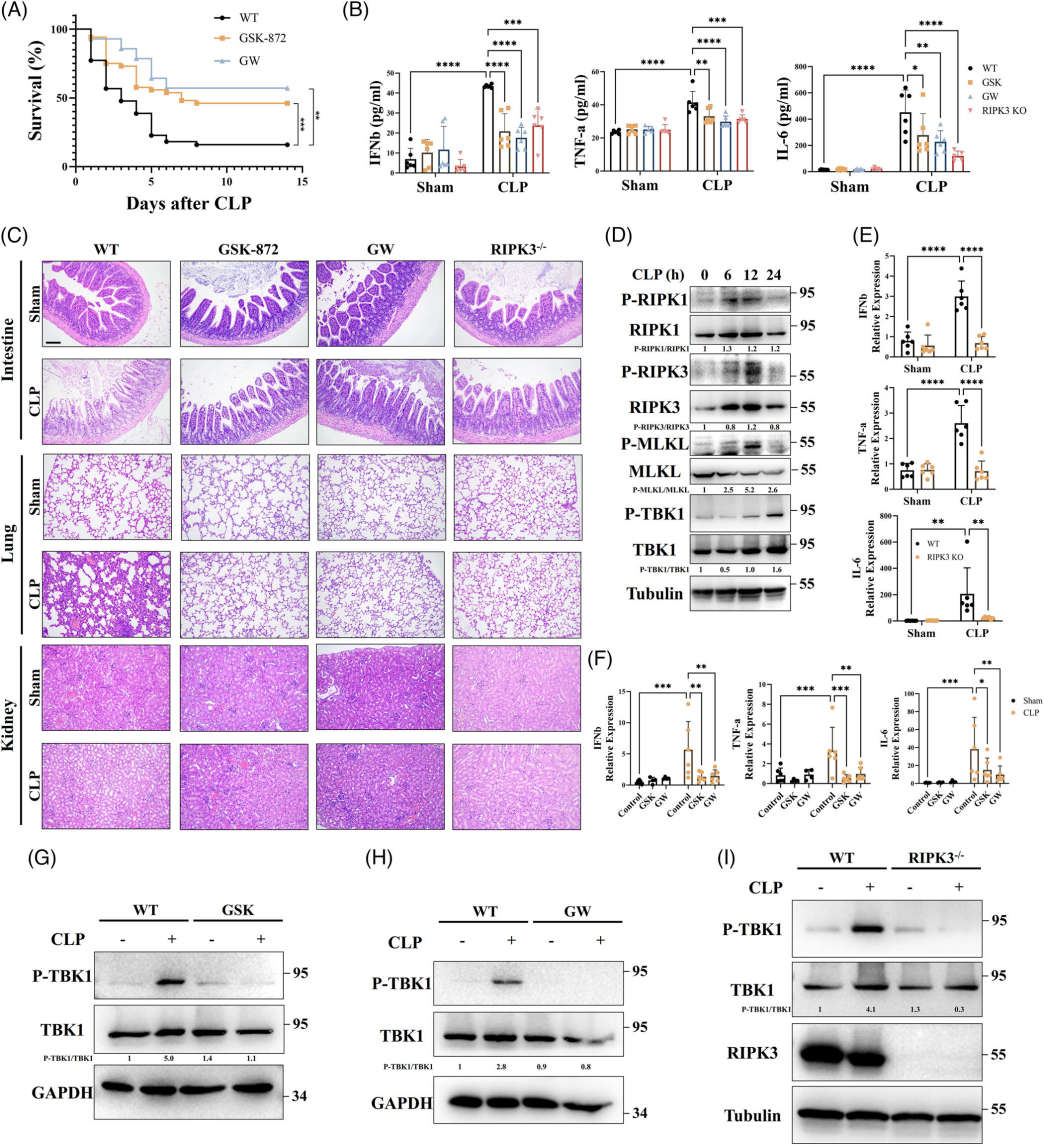
Inhibition of necroptotic signalling protects against lethal sepsis. (A) Animal survival was monitored after CLP in WT mice treated with GSK (3 mg/kg) or GW (2 mg/kg). (B) Plasma IFNβ, TNF‐α and IL‐6 were tested by ELISA. (C) Representative images of H&E staining of intestine, lung and kidney in RIPK3^−/−^ and WT mice treated with GSK (3 mg/kg) or GW (2 mg/kg) after CLP for 24 h. (D) Intestines was collected in WT mice after CLP at 0 h, 6 h, 12 h and 24 h. STING signalling and necroptosis in intestines were detected by western blot. The experiment was repeated at least three times. (E) qPCR analysis of IFNβ, TNF‐α and IL‐6 mRNA in murine intestines of WT and RIPK3^−/−^ mice after CLP for 24 h. (F) qPCR analysis of IFNβ, TNF‐α and IL‐6 mRNA in murine intestines of WT treated with GSK (3 mg/kg) or GW (2 mg/kg) after CLP for 24 h. (G–I) Western blot was used to detect STING signalling in intestines of RIPK3^−/−^ mice or WT treated with GSK (3 mg/kg) or GW (2 mg/kg) after CLP for 24 h. The experiment was repeated at least three times. Scale bars = 1 µm. Data were shown as the mean ± SD. *p < 0.05, **p < 0.01, ***p < 0.001, ****p < 0.0001.

We next sought to clarify whether necroptotic signalling affects multiple organ dysfunction and STING signalling during sepsis. An in vivo sepsis model was established by cecal ligation and puncture (CLP). Notably, administration of GSK and GW significantly ameliorated CLP‐induced lethality (Figure 9A). Furthermore, inhibition of necroptotic signalling or RIPK3 knockout significantly decreased IFNβ and inflammatory cytokines in circulation (Figure 9B) and attenuated damage to intestine, lung and kidney (Figure 9C) after CLP. CLP enhanced IFNβ expression in the intestine, which was dependent on STING signalling (Figure S7A). We sought to determine changes in STING and necroptotic signalling after CLP. We demonstrated that both STING and necroptotic signalling were triggered in the small intestine after CLP (Figures 9D and S8A). Moreover, RIPK3 knockout and treatment with GSK or GW significantly restrained IFNβ and inflammatory cytokines at transcription level in the intestine (Figures 9E and F). In addition, necroptosis deficiency suppressed STING signalling in the intestines after CLP (Figures 9G–I). Taken together, these results confirm that necroptotic signalling orchestrating STING signalling exacerbates sepsis‐induced multiple organ dysfunction and systemic inflammation.

## 3 DISCUSSION

**FIGURE 10:**
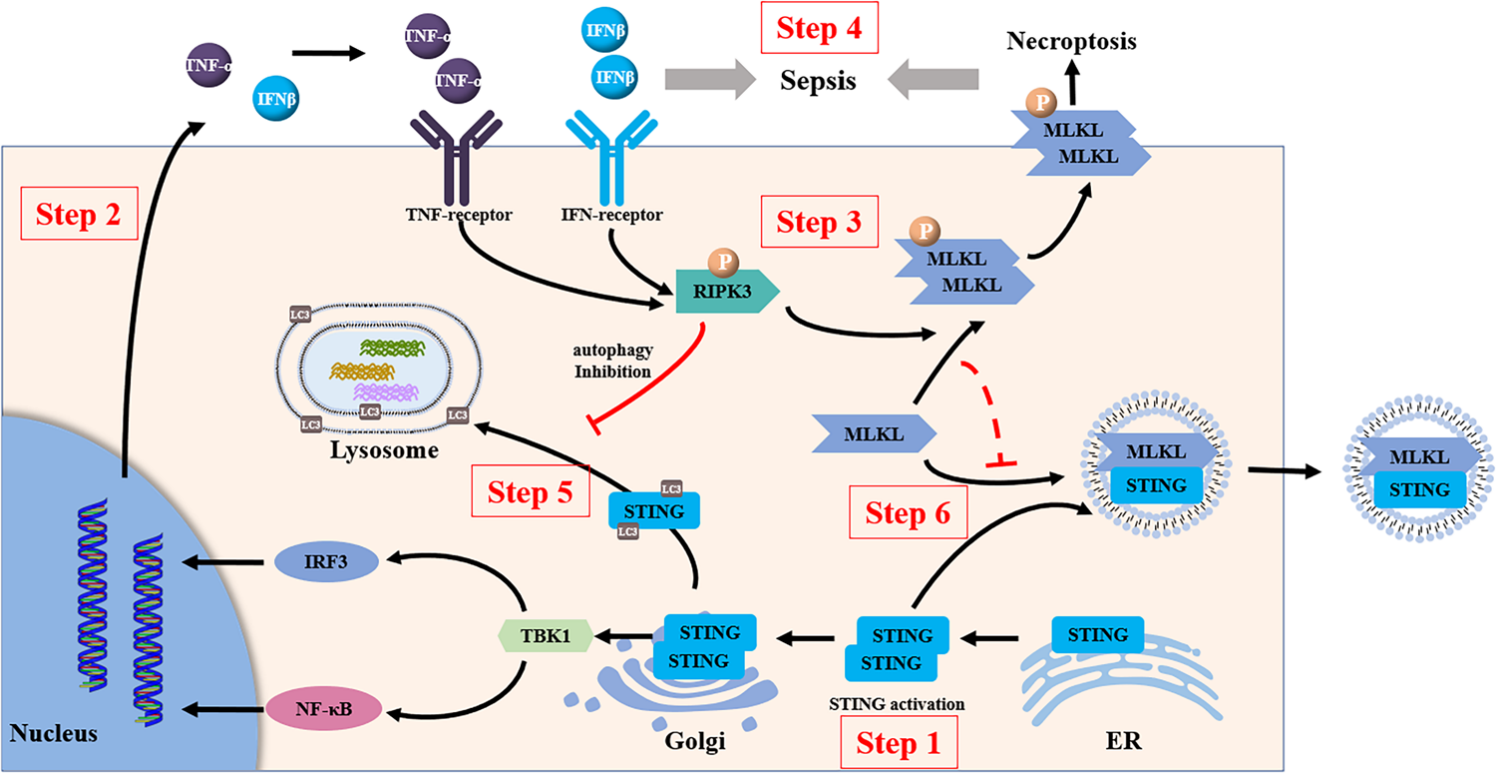
Schematic representation of crosstalk between STING signalling and necroptotic signalling. Step1, upon STING activation, activated STING traffics from the endoplasmic reticulum (ER) to the Golgi and recruits TBK1, subsequently triggering IRF3 and NF‐κB signalling. Step2, STING‐mediated inflammatory cytokines, including IFNβ and TNF‐α, are released outside cells and activates receptors of IFN and TNF‐α. Step3 and Step4, necroptotic signalling is triggered in response to IFNβ and TNF‐α and results in sepsis. Step5, autophagy of STING is the negative feedback of STING signalling. However, RIPK3 participates in inhibiting autophagic flux and sustains STING signalling. Step6, intrinsic MLKL binding to activated STING is secreted outside cells, which is restrained by the pro‐necroptotic activity of MLKL.

Our recent study observed an association between STING signalling and necroptosis in acute intestinal injury.^13^ Further experiments revealed that necroptosis is not limited to the downstream effects of STING signalling. Surprisingly, inhibition of RIPK3 and MLKL restrained STING signalling. Therefore, STING‐mediated necroptosis is associated with a positive feedback mechanism that maintains STING activation (Figure 10).

RIPK3 phosphorylation is a prerequisite for MLKL activation during necroptosis. However, increasing evidence has shown that RIPK3 plays a cell death‐independent role in the production of cytokines and chemokines by inducing intrinsic inflammatory signalling, including NF‐κB, MAPK and NLRP3 inflammasome signalling.^11^ RIPK3 regulates mitochondrial metabolism^21^ and autophagy.^22^, ^23^ In this study, we demonstrated that RIPK3 knockout in HT‐29 cells and BMDM significantly suppressed STING signalling, independent of RIPK1 and MLKL. Comparative pathway enrichment analysis of proteome profiling using GO revealed that autophagosome maturation was a key change between the WT and RIPK3^−/−^ intestines under DMXAA stimulation. Further experiments showed that RIPK3 overexpression attenuated STING autophagy and RIPK3 knockout induced a decrease in P62 and an increase in LC3‐II, whereas suppression of autophagy alleviated the inhibitory effect of RIPK3 knockout on STING signalling. Moreover, the kinase domain of RIPK3 was found to be responsible for regulation of STING signalling, and inhibition of RIPK3 kinase activity promoted autophagy flux induced by STING activation.

RIPK3 positively regulates autophagy through its association with AMP‐activated protein kinase (AMPK) and unc‐51 like autophagy activating kinase 1 (ULK1).^22^, ^23^ Interestingly, the role of AMPK and ULK1 in STING autophagy remains controversial.^4^, ^24^ Moreover, necroptosis triggers early autophagy via RIPK3, but inhibits late autophagy.^23^ Thus, available evidence only suggests that RIPK3 initiates early autophagy.^22^, ^23^ In this study, we observed that RIPK3 blocked autophagic flux after STING activation, which is independent of necroptotic activity of RIPK3. RIPK3 blocking autophagy in STING is vital for sustaining STING signalling.

MLKL is a terminal effector of necroptosis. Recent attention has focused on the necroptosis‐independent functions of MLKL.^12^ Intriguingly, we found that inhibition of MLKL‐mediated membrane perturbation with NSA or GW806742X restrained STING signalling. MLKL blocks autophagy and consequently contributes to non‐alcohol‐related fatty liver disease.^25^ However, MLKL overexpression significantly suppressed STING protein levels, which were not affected by inhibition of autophagy. GO analysis of proteome profiles revealed that STING activation was associated with vesicle‐mediated transport. Moreover, in addition to autophagy, MLKL participates in vesicle‐mediated transport and the generation of extracellular vesicles.^18^, ^26^ Further experiments indicated that activated STING bound to MLKL underwent extracellular secretion, which was intensified by abrogation of the pro‐necroptotic activity of MLKL. Interestingly, it has been reported that MLKL‐mediated extracellular vesicle generation relies on phosphorylation of T357/S358 in MLKL,^18^ whereas MLKL–T357A/S358A mutants still suppress STING signalling. Meanwhile, neither the pseudokinase domain nor the four‐helix bundle domain of MLKL inhibited STING signalling, similar to previous studies showing that the four‐helix bundle domain of MLKL does not enhance generation of extracellular vesicles.^18^ In addition, MLKL deficiency significantly enhances STING signalling. Overall, intrinsic MLKL exerts negative effects in STING signalling. Thus, STING‐mediated necroptosis represents a positive feedback mechanism, that triggers MLKL pro‐necroptotic activity, decreases STING release and sustains STING signalling.

Studies on RIPK3/MLKL signalling have largely focused on necroptosis. Knockout of necroptotic signalling is thought to inhibit cell death and downstream inflammation. However, the non‐canonical roles of RIPK3 and MLKL are often ignored. RIPK3 or MLKL knockouts may affect other inflammatory pathways. To date, knockout of necroptotic signalling reveals a controversial role in sepsis.^27^, ^28^ In this study, we demonstrated that administration of GSK and GW inhibited both cell death and STING signalling in sepsis, subsequently ameliorating sepsis‐induced multiple organ dysfunction and systemic inflammation. These findings suggest that inhibitors of necroptotic signalling can be extended to suppression of STING and treatment of sepsis.

## 4 MATERIALS AND METHODS

### 4.1 Patients and specimens

The collection of human blood samples and intestinal tissue was from our previous study.^13^ Sepsis and septic shock were defined according to previous reports.^19^ PBMC isolation was performed using Lymphoprep (07801; STEMCELL) and SepMateTM tube (85415; STEMCELL) according to the manufacturer's instructions.

### 4.2 Mice and animal experiments

WT, RIPK3^−/−^ and STING^−/−^ C57BL/6 mice were purchased from Model Animals Research Center of Nanjing University. MLKL^−/−^ mice were gifts from Dr. Jiahuai Han (Xiamen University, China). Eight‐ to ten‐week‐old mice were used for the experiments.

CLP model was conducted to generate a sepsis model as described previously.^8^ Mice received an intraperitoneal injection of 3 mg/kg GSK‐872 (MedChemExpress) or 2 mg/kg GW806742X (MedChemExpress) dissolved in SBE‐β‐CD solution at 0, 12, 24, 48 and 72 h after CLP.

DMXAA (HY‐10964; MedChemExpress) was dissolve in 7.5% sodium bicarbonate solution for animal experiments. Mice received an intraperitoneal injection of 25 mg/kg DMXAA and 6 h later experiments were terminated for tissue collection. Control group was injected with 7.5% sodium bicarbonate solution without DMXAA.

### 4.3 Cell culture, BMDM isolation and cell stimulation

The HT‐29 cells (ATCC) were cultured in McCoy's 5A Medium Modified (Servicebio) supplemented with 10% foetal bovine serum (FBS; Gibco), penicillin and streptomycin. RAW‐Lucia™ ISG Cells, RAW‐Lucia™ ISG‐KO‐TBK1 Cells and RAW‐Difluo™ mLC3 Cells were purchased from InvivoGen and cultured in Dulbecco's modified Eagle's medium (DMEM; Servicebio) with 10% FBS, penicillin and streptomycin. HEK293T cells and THP‐1 (ATCC) were cultured in DMEM with 10% FBS, penicillin and streptomycin. Less than 20 generations of cells were used in the experiment. The levels of IFN‐luciferase reporter were monitored using QUANTI‐Luc™ (InvivoGen).

BMDMs were isolated from the bone marrow of the tibia and femur. BMDMs were washed, resuspended and cultured in DMEM with 10 ng/mL CSF‐1 (315‐03; PeproTech), 10% FBS, penicillin and streptomycin for 7 d.

ADU‐S100 (HY‐12885B), DMXAA (HY‐10964), necrostatin‐1 (HY‐15760), GSK‐872 (HY‐101872), NSA (HY‐100573), GW806742X (HY‐112292), Z‐VAD‐FMK (HY‐16658B), c‐di‐AMP (HY‐12326), Bafilomycin A1 (HY‐100558) and 2′,3′‐cGAMP (HY‐100564) were purchased from MedChemExpress. ADU‐S100, DMXAA, c‐di‐AMP, 2′,3′‐cGAMP and Bafilomycin A1 were applied to RAW264.7 cells at 13.5 µM, 50 µg/mL, 20 µM, 20 µM and 100 nM, respectively. ADU‐S100 was applied to HT‐29 cells at 27 µM. Bafilomycin A1 and NSA were applied to HEK293T at 100 nM and 10 µM, respectively. ADU‐S100 and NSA were applied to THP‐1 cells at 13.5 and 10 µM, respectively.

### 4.4 Plasmid transfection and siRNA transfection

According to the manufacturer's instructions, all plasmid and siRNA were transfected with Lipofectamine‐3000 (Invitrogen). Targeting sequences of siRNA for human MLKL were 5′‐GGUGUGAAGAGAUGAAAUATT‐3′ and 5′‐UAUUUCAUCUCUUCACACCTT‐3′. Targeting sequences of siRNA for mouse MLKL were 5′‐GGAAUUGUACUCUGGGAAATT‐3′ and 5′‐UUUCCCAGAGUACAAUUCCTT‐3′.

### 4.5 Generation and validation of RIPK3 knockout HT‐29 cells

Three Single‐guide RNA targeting the human RIPK3 gene (5′‐CAGTGTTCCGGGCGCAACAT‐3′, 5′‐CGCCTTTGCCGACGAGCTCC‐3′ and 5′‐GAATTCGTGCTGCGCCTAGA‐3′) was cloned into GenCRISPR eSp Cas9 Puro Plasmid (GenScript), respectively. HT‐29 cells were transfected with three above‐mentioned plasmid DNA via electroporation and then selected with 1 µg/mL puromycin for 7 days. Then, single‐colony was selected to identify RIPK3 knockout cells, which was validated by immunoblotting.

### 4.6 Immunoprecipitation and immunoblotting

Cells were treated with cell lysis buffer for Western and IP (Beyotime). The indicated antibodies were added to cell lysates or supernatant, followed by incubation with gentle rocking overnight at 4°C. Then, protein G agarose beads were added to cell lysates, which was incubated with gentle rocking for 4 h at 4°C. Next, samples were eluted in SDS‐PAGE sample loading buffer and boiled. For immunoprecipitation, anti‐FLAG (AE005; ABclonal), STING (19851‐1‐AP; Proteintech) and MLKL (PA5‐102810; Invitrogen) antibodies were used.

The primary antibodies were used including STING (19851‐1‐AP; Proteintech), hp‐STING (S366, 50907; CST), mp‐STING (S365, 72971; CST), p‐TBK1 (S172; CST), TBK1 (38066; CST), IRF3 (4302; Cell Signaling), p‐IRF3 (S396, 29047; CST), mp‐RIPK3 (T231+S232, ab222320; Abcam), mp‐MLKL(S345, ab196436; Abcam), hp‐RIPK3 (S227, ab209384; Abcam), hp‐MLKL (S358, ab187091; Abcam), hMLKL (A19685; ABclonal), mMLKL (37705; CST), hRIPK3 (86671; CST), mRIPK3 (15828; CST), mp‐RIPK1 (S166, 31122; CST), RIPK1 (3493; CST), LC3 (12741; CST), P62 (A7758; ABclonal), β‐actin (4970; CST), β‐Tubulin (AC021; ABclonal), GAPDH (60004‐1‐Ig; Proteintech), FLAG‐tag (AE063; ABclonal), HA‐tag (ab236632; Abcam), MYC‐tag (AE070; ABclonal) and GFP‐tag (AE012; ABclonal).

### 4.7 RNA isolation and quantitative real‐time PCR analysis

Total RNA was extracted by employing TRIzol reagent (Invitrogen). RNA was reversely transcribed into cDNA via a reverse transcriptase kit (R223‐01; Vazyme). Quantitative real‐time PCR was performed via using SYBR qPCR Master Mix (Vazyme). The relevant sequences of primers for quantitative real‐time PCR analyses are listed in Table S2.

### 4.8 ELISA

The protein levels of murine IFN‐β (RK00420; ABclonal), murine IL‐6 (KE10007; Proteintech) and murine TNF‐α (RK00027; ABclonal) in the plasma were tested via commercially ELISA kits.

### 4.9 Histology, immunohistochemistry and immunofluorescence

Histology, immunohistochemistry and immunofluorescence for tissue or cell were performed as previously described.^13^ Primary antibodies were used including hp‐RIPK3 (S227, ab209384; Abcam), hp‐MLKL (S358, ab187091; Abcam), STING (19851‐1‐AP; Proteintech), LC3 (12741; CST) and FLAG‐tag (AE063; ABclonal). Nucleus was stained with DAPI.

### 4.10 Purification of supernatant protein

Protein was purified from cell culture supernatant by using Amicon® Ultra‐4 Centrifugal Filter Unit (UFC8010, Millipore) according to the manufacturer's instructions.

### 4.11 Proteome profiling

Firstly, the samples were conducted for lytic treatment for protein extraction. Protein was quantified according to BCA method and an appropriate amount of protein was taken from each sample for trypsin enzymolysis using FASTA method. The peptide was freeze‐dried and re‐dissolved with 40 µL dissolution buffer, and the peptide was quantified. 100 µg peptide was taken from each sample and labelled according to the instructions of TMT labelling Kit (ThermoFisher).

The labelled peptides were pooled and graded via an AKTA Purifier 100. The products were chromatographically separated and analysed by mass spectrometry.

Data of protein expression was imported into R (R4.2.1), and PCA analysis was performed by using mixomics (6.22). The value of R2X is 0.749 (PC1 = 0.4294373, PC2 = 0.3198248). Under ideal conditions, in PCA, inter‐group samples should be dispersed and intra‐group samples should be clustered together.

The heatmap was drawn by importing data of proteome into R (R4.2.1) and using pheatmap (1.0.12). *Z*‐score of data was normalised. GO analysis showing functional enrichment analysis of differentially expressed protein was conducted by Clusterprofiler (version4.6.0) and R (R4.2.1). First, Clusterprofiler (version4.6.0) software was used to make GO annotation for proteins. Then, R (R4.2.1) was used for each GO classification, GO annotation and the pathway enrichment.

### 4.12 Statistical analysis

Comparing differences between groups were conducted via Student's *t*‐test or one‐way ANOVA. *p* < 0.05 was considered for statistical significance. Statistical analyses were performed via GraphPad Prism software 9.0.

## CONFLICT OF INTEREST STATEMENT

The authors declare no conflict of interest.

## FUNDING INFORMATION

National Natural Science Foundation of China (82072149, 82072223 and 82100597), Natural Science Foundation of Jiangsu Province (BK20201116 and BK20210039) and Medical Scientific Research Project of Jiangsu Health Committee (ZDB2020028).

## Supporting information

SUPPORTING INFORMATION

SUPPORTING INFORMATION

SUPPORTING INFORMATION

## Data Availability

Proteome data were deposited to the ProteomeXchange Consortium (http://proteomecentral.proteomexchange.org) via the iProX partner repository using the dataset identifier, PXD034614. Data supporting the findings of this study are available from the corresponding author upon reasonable request.
