# Supplementary material for: RIPK3–MLKL necroptotic signalling amplifies STING pathway and exacerbates lethal sepsis
Source: Clin Transl Med. 2023 Jul 20;13(7):e1334. doi: 10.1002/ctm2.1334 (PMC10359592; doi:10.1002/ctm2.1334)
Supplement: Supplementary file 1 — SUPPORTING INFORMATION [file CTM2-13-e1334-s003.docx]

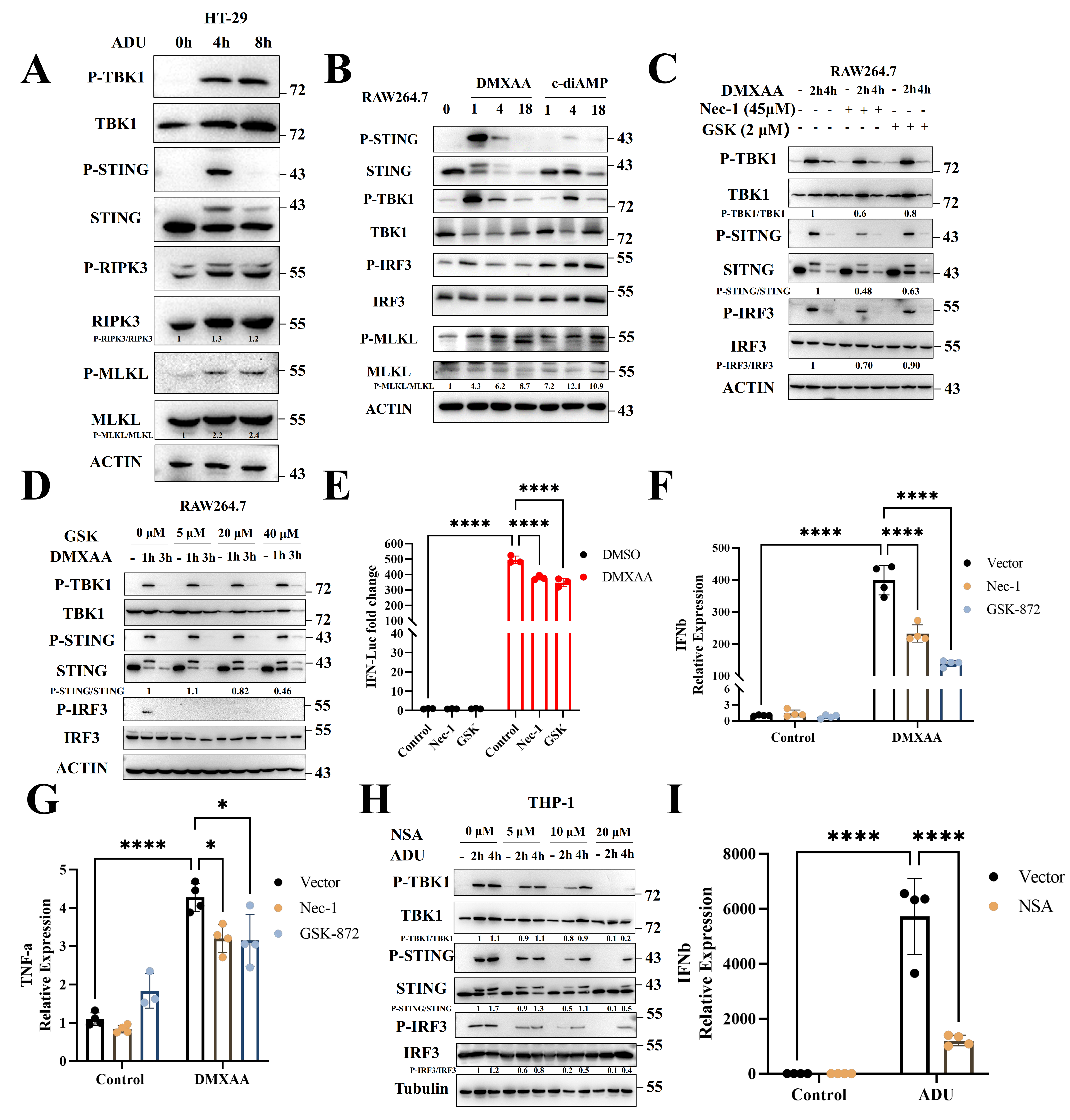


**Supplementary Fig.1 Inhibition of necroptotic signaling restrains activation of STING signaling.**

A Immunoblot analysis of STING and necroptotic signaling in HT-29 cells under ADU stimulation (27 μM). B Immunoblot analysis of STING and necroptotic signaling in RAW264.7 cells under DMXAA (50 μg/ml) or c-diAMP (20 μM) stimulation. C Western blot analysis of STING signaling in RAW264.7 cells treated with inhibitors of necroptotic signaling (Nec-1 and GSK) and DMXAA (50 μg/ml). The experiment was repeated at least three times. D Assessment of STING signaling treated with GSK in a dose-dependent manner under DMXAA (50 μg/ml) stimulation in RAW264.7 cells. The experiment was repeated at least three times. E Assessment of STING–mediated IFN luciferase reporter activation in RAW264.7 cells treated with inhibitors of necroptotic signaling (30 μM Nec-1 and 5 μM GSK) and DMXAA (50 μg/ml). F and G qPCR analysis of IFNβ and TNF-α mRNA in RAW264.7 cells treated with inhibitors of necroptotic signaling (30 μM Nec-1 and 5 μM GSK) and DMXAA (50 μg/ml). H Assessment of STING signaling treated with NSA in a dose-dependent manner under ADU (13.5 μM) stimulation in THP-1 cells. The experiment was repeated at least three times. I qPCR analysis of IFNβ mRNA in THP-1 cells treated with NSA (10 μM) and ADU (13.5 μM). Data were shown as the mean ± SD. *P < 0.05, **P < 0.01, ***P < 0.001, ****P < 0.0001.


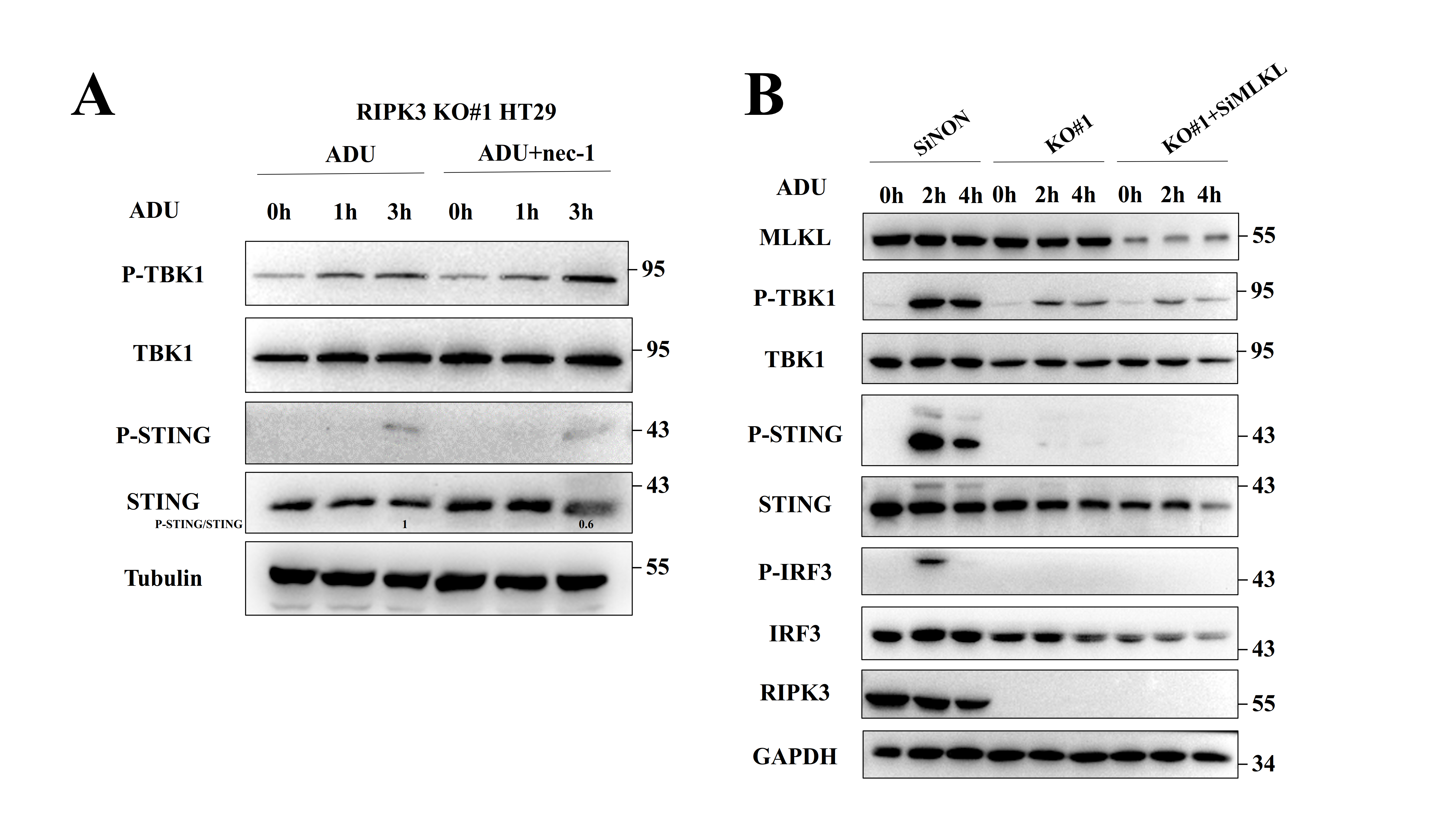


**Supplementary Fig.2 Nec-1 and knockdown of MLKL are dispensable for inhibition of STING signaling induced by RIPK3 knockout in HT-29 cells**

A Immunoblot analysis of STING signaling in RIPK3 knockout HT-29 cells under ADU stimulation (27 μM) and Nec-1 (45 μM). B Immunoblot analysis of STING signaling in WT and RIPK3 knockout and MLKL knockdown HT-29 cells under ADU stimulation (27 μM).


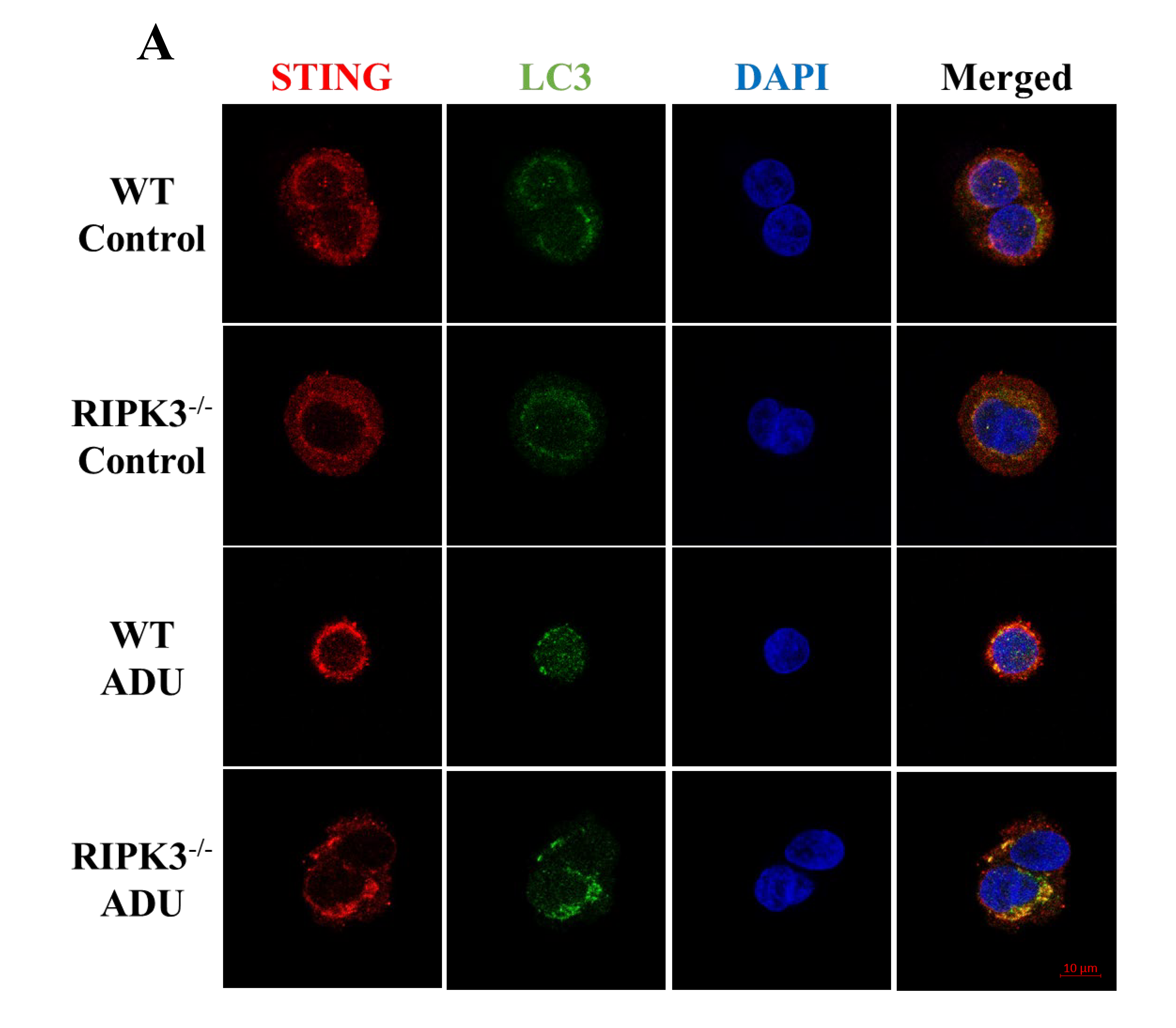


**Supplementary Fig.3 RIPK3 knockout promotes the interaction between STING and LC3.**

A Confocal microscopy analysis of STING and LC3 in WT and RIPK3 knockout HT-29 cells after ADU (27 μM) stimulation. Nuclear DNA was labeled using DAPI.


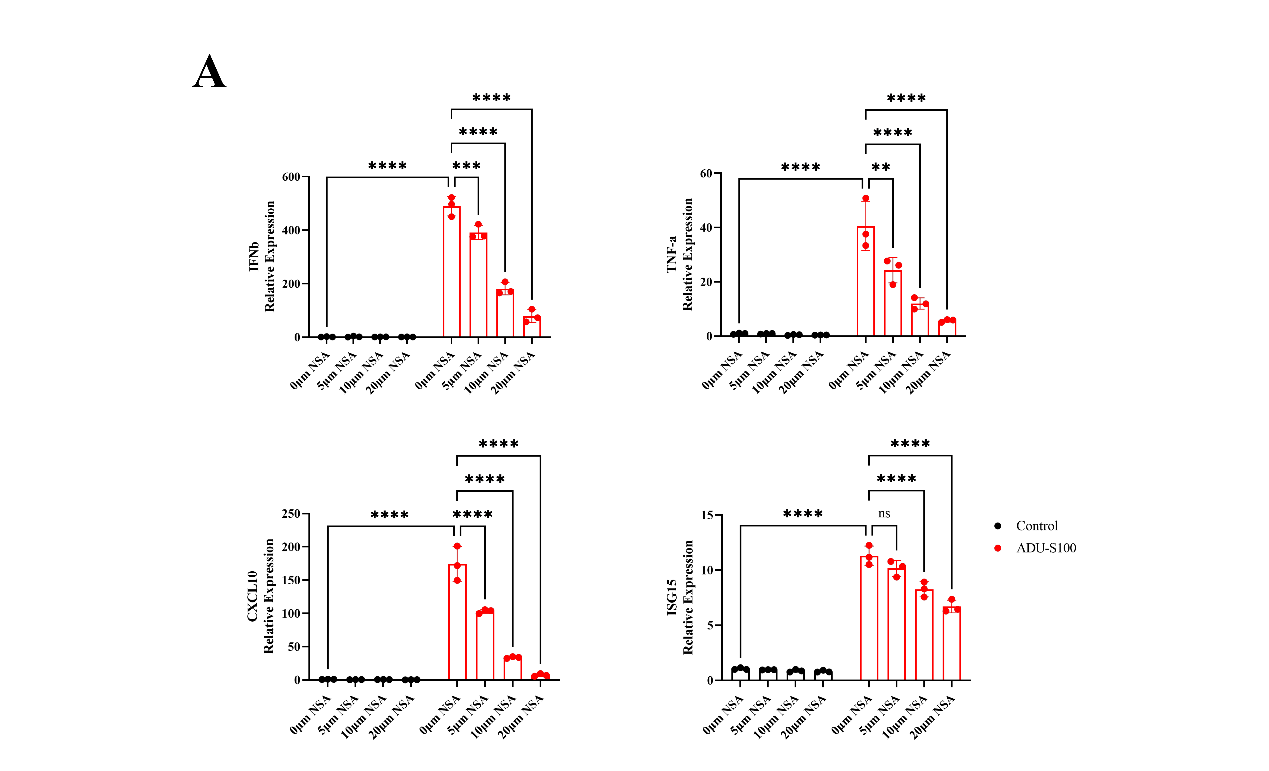


**Supplementary Fig.4 NSA restrains expression of inflammatory cytokines induced by ADU-S100**

A qPCR analysis of IFNβ, TNF-α, CXCL10, and ISG15 in HT-29 cells stimulated with ADU (27 μM) and NSA in a dose-dependent manner. Data were shown as the mean ± SD. *P < 0.05, **P < 0.01, ***P < 0.001, ****P < 0.0001.


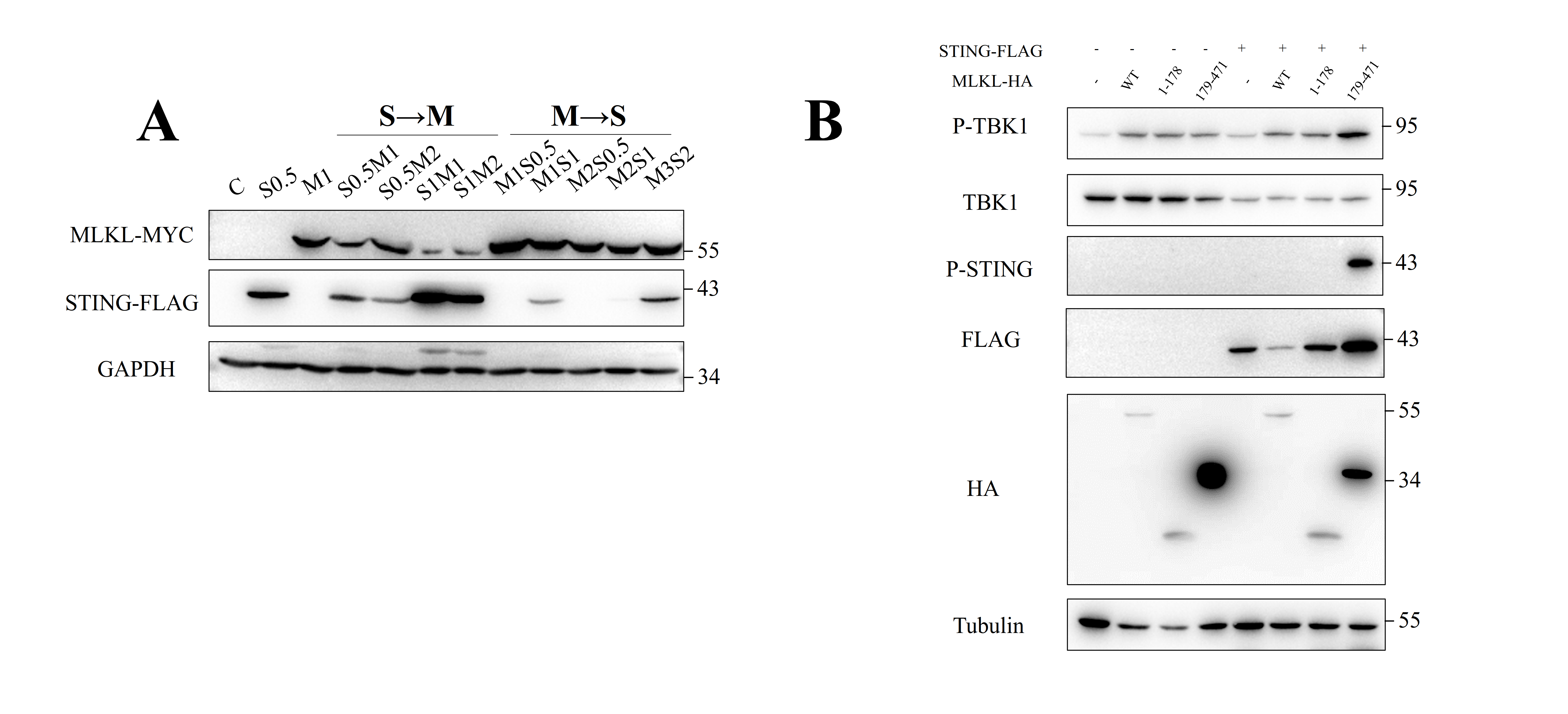


**Supplementary Fig.5 MLKL restrains expression of STING in a dose-dependent manner**

A Different dose (0.5 - 3 μg) of STING plasmid and MLKL plasmid were expressed in HEK293T cells at 4 h interval. Western blot was performed to analyze indicated protein. B Immunoblot analysis of STING signaling in HEK293T cells co-expressed with STING plasmid and MLKL plasmid. The experiment was repeated at least three times.


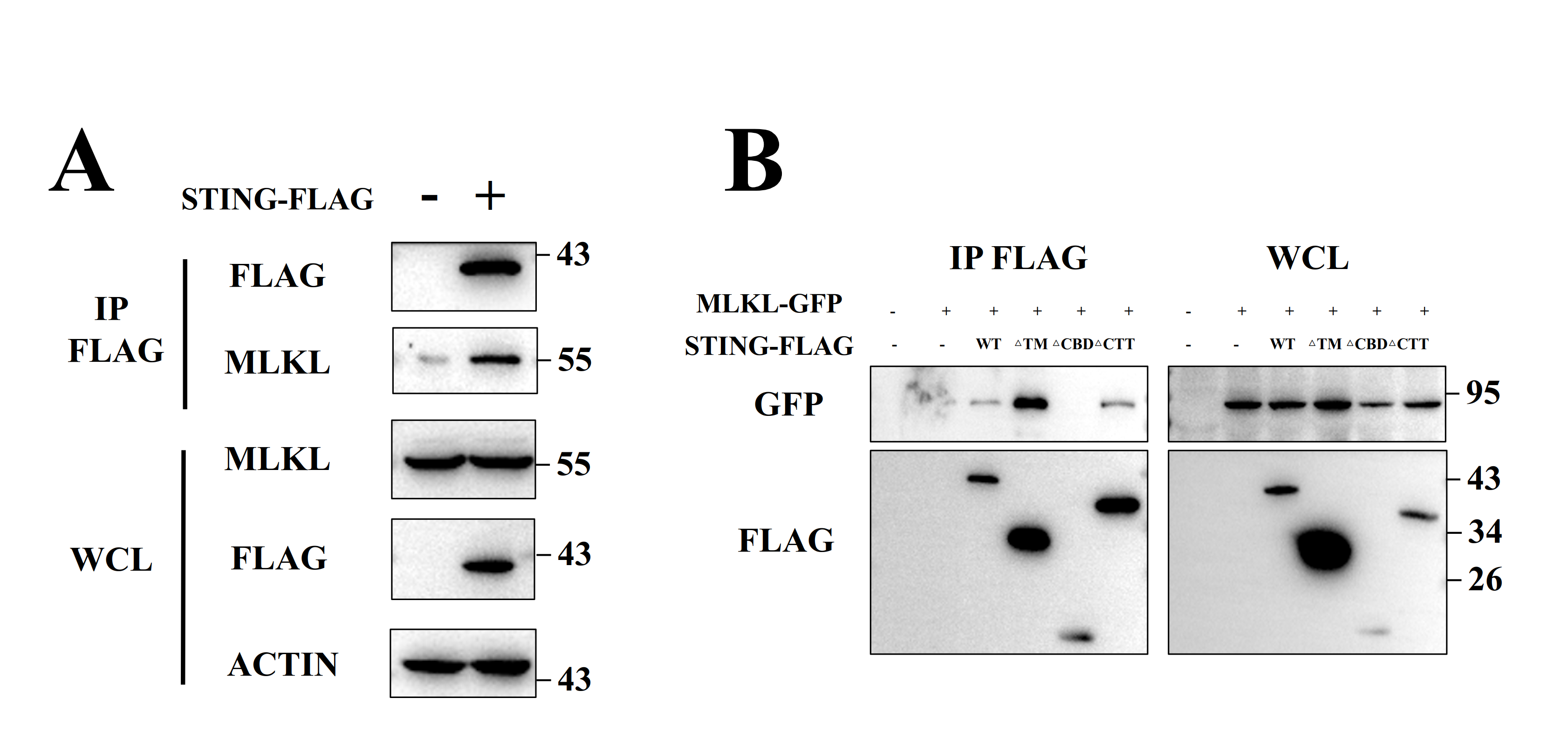


**Supplementary Fig.6 STING binds MLKL during activation of STING**

A STING plasmid was expressed in HEK293T cells. The cell lysates were immunoprecipitated using anti-STING antibody and then analyzed by immunoblotting. The experiment was repeated at least three times. B MLKL plasmid was expressed in HEK293T cells after transfection of STING mutant plasmid (TM domain-deleted STING, △TM; CBD domain-deleted STING, △CBD; CTT domain-deleted STING, △CTT) at 4 h interval. The cell lysates were immunoprecipitated using anti-FLAG antibody and then analyzed by immunoblotting. The experiment was repeated at least three times.


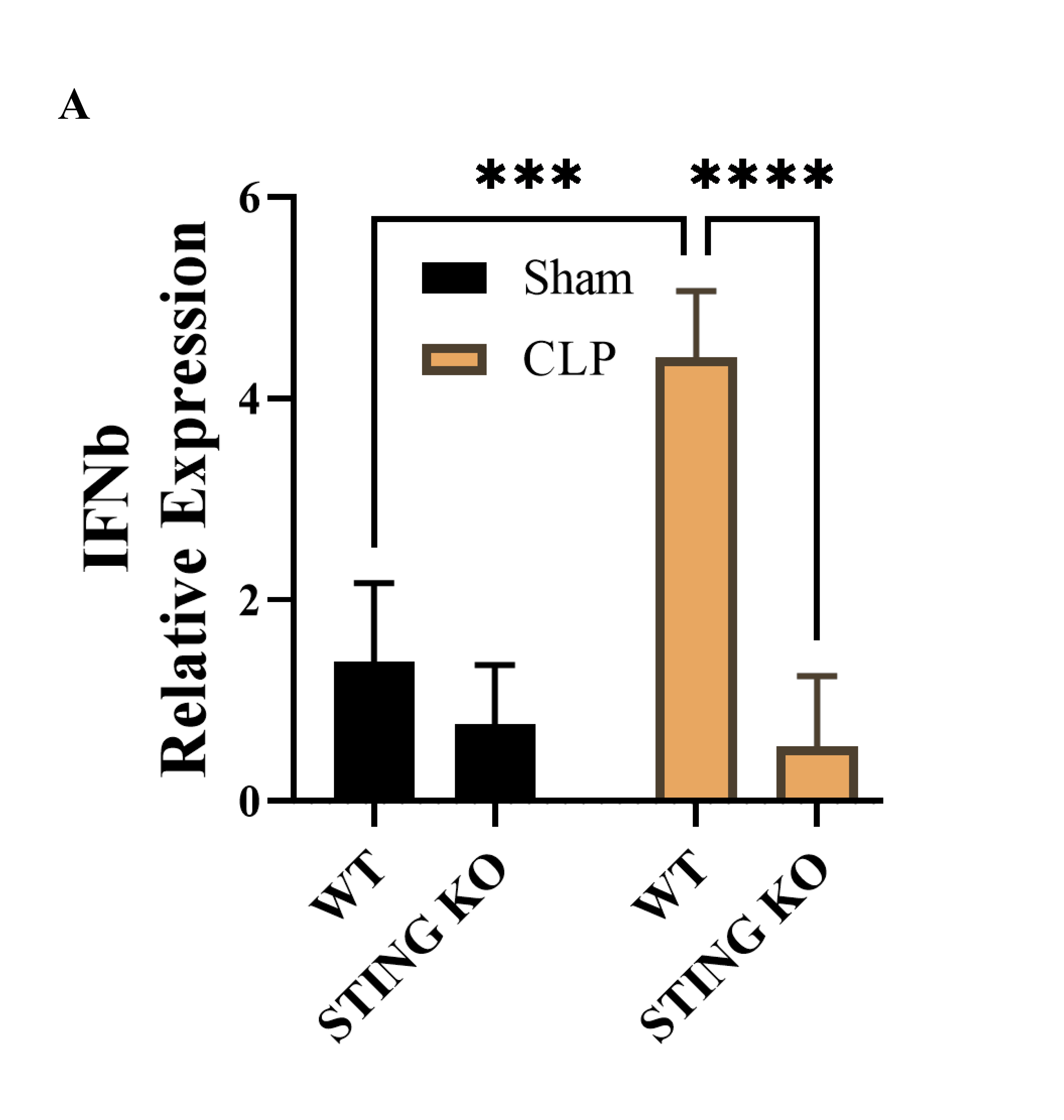


**Supplementary Fig.7 STING contributes to expression of IFN**β **in sepsis.**

A qPCR analysis of IFNβ mRNA in intestines of WT and STING^-/-^ mice after CLP for 24h. Scale bars = 1 μm. Data were shown as the mean ± SD. *P < 0.05, **P < 0.01, ***P < 0.001, ****P < 0.0001.


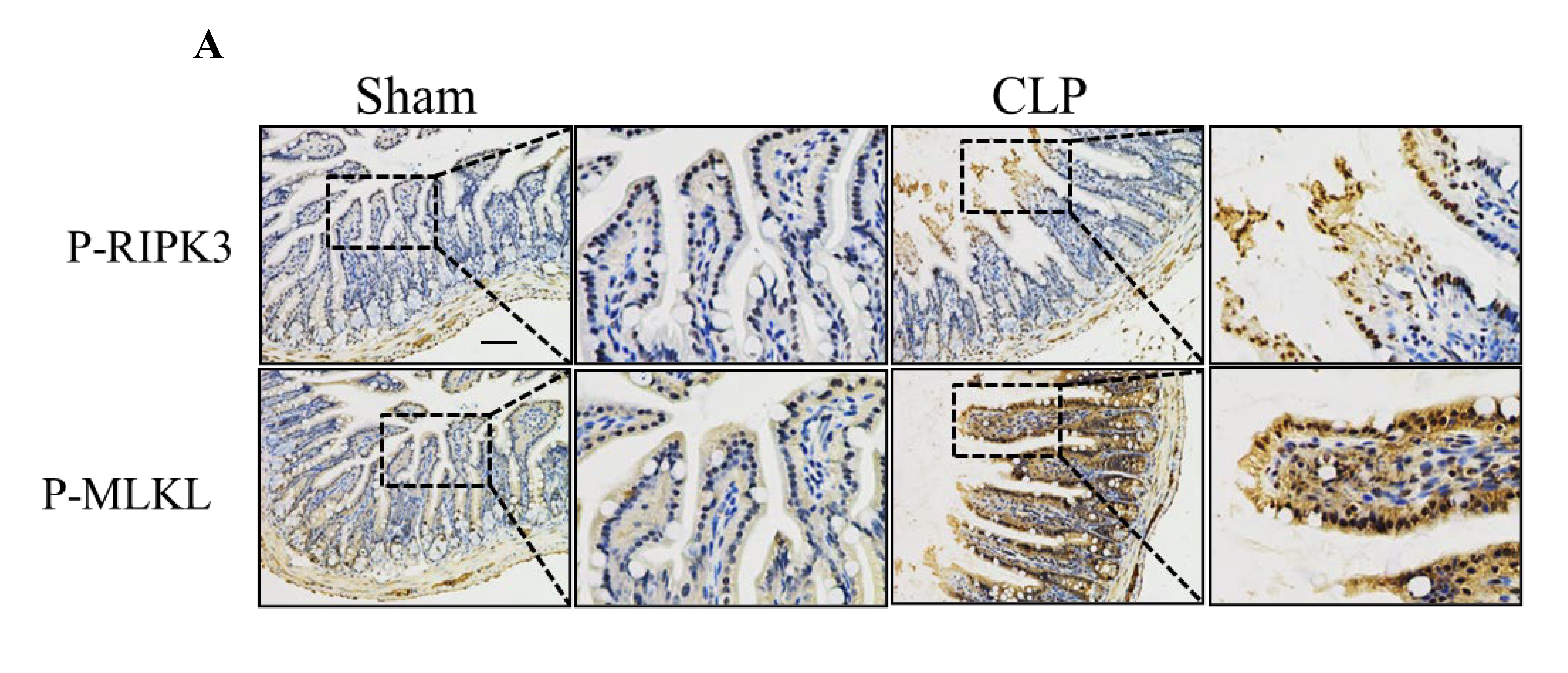


**Supplementary Fig.8 Necroptosis occurs in the intestinal mucosa after CLP.**

A Representative images of immunohistochemistry for P-RIPK3 and P-MLKL in intestinal tissue after CLP. Scale bars = 1 μm.
